# Mapping the chemotactic landscape in NK cells reveals subset-specific synergistic migratory responses to dual chemokine receptor ligation

**DOI:** 10.1016/j.ebiom.2023.104811

**Published:** 2023-09-21

**Authors:** Mieszko Lachota, Katarzyna Zielniok, Daniel Palacios, Minoru Kanaya, Leena Peena, Hanna Julie Hoel, Merete Thune Wiiger, Lise Kveberg, Wojciech Hautz, Radosław Zagożdżon, Karl-Johan Malmberg

**Affiliations:** a Department of Clinical Immunology, Medical University of Warsaw, Warsaw, Poland; b Department of Ophthalmology, Children’s Memorial Health Institute, Warsaw, Poland; c Department of Cancer Immunology, Institute for Cancer Research, Oslo University Hospital, University of Oslo, Norway; d Finnish Red Cross Blood Service, Research and Development, Helsinki, Finland; e Center for Infectious Medicine, Department of Medicine Huddinge, Karolinska Institutet, Stockholm, Sweden

**Keywords:** NK cells, Natural killer cells, Cancer, Immunology, Migration, Chemokine receptors, Synergy, Differentiation, Immunotherapy, Cell therapy, TCGA, Adoptive therapy

## Abstract

**Background:**

Natural killer (NK) cells have a unique capability of spontaneous cytotoxicity against malignant cells and hold promise for off-the-shelf cell therapy against cancer. One of the key challenges in the field is to improve NK cell homing to solid tumors.

**Methods:**

To gain a deeper understanding of the cellular mechanisms regulating trafficking of NK cells into the tumor, we used high-dimensional flow cytometry, mass cytometry, and single-cell RNA-sequencing combined with functional assays, creating a comprehensive map of human NK cell migration phenotypes.

**Findings:**

We found that the chemokine receptor repertoire of peripheral blood NK cells changes in a coordinated manner becoming progressively more diversified during NK cell differentiation and correlating tightly with the migratory response of the distinct NK cell subsets. Simultaneous ligation of CXCR1/2 and CX3CR1, synergistically potentiated the migratory response of NK cells. Analysis of 9471 solid cancers from publicly available TCGA/TARGET repositories revealed dominant chemokine patterns that varied across tumor types but with no tumor group expressing ligands for more than one chemokine receptor present on mature NK cells.

**Interpretation:**

The finding that chemokine stimulation can elicit a synergistic migratory response in NK cells combined with the identified lack of naturally occurring pairs of chemokines-chemokine receptors in human cancers may explain the systematic exclusion of NK cells from the tumor microenvironment and provides a basis for engineering next-generation NK cell therapies against malignancies.

**Funding:**

The Polish Ministry of Science and Higher Education, the National Science Centre, Poland, 10.13039/100008730The Norwegian Cancer Society, the Norwegian Research Council, the South-Eastern Norway Regional Health Authority, The Swedish Cancer Society, the Swedish Children's Cancer Foundation, The Swedish Research Council, The Center of Excellence: Precision Immunotherapy Alliance, Knut and Alice Wallenberg Foundation and 10.13039/100000054National Cancer Institute.

**Research in context**

**Evidence before this study**

Natural killer (NK) cells hold promise as a highly functional template for genetic engineering in the development of next generation off-the-shelf cell therapies. However, a prerequisite for extending the success of cell therapy to solid tumors is that the cells reach the intended target organ. In contrast to T cells, highly differentiated NK cells are rarely found in solid tumors and trafficking patterns of NK cells remain poorly understood. Furthermore, there is limited information on the dynamic regulation of chemokine receptor expression during NK cell differentiation and *ex vivo* expansion for cell therapy.

**Added value of this study**

In this study, we investigated the trafficking patterns of human NK cells utilizing high-dimensional flow cytometry, mass cytometry, and single-cell RNA-sequencing combined with functional assays. We found that the chemokine receptor repertoire of peripheral blood NK cells changes in a coordinated fashion becoming gradually more diversified during the differentiation process. The chemokine receptor expression correlated tightly with the migratory response of the distinct NK cell subsets. We also found that simultaneous ligation of CXCR1/2 and CX3CR1 receptors led to a synergistically enhanced migratory response. Investigation of 9471 solid cancer cases in the TCGA/TARGET databases revealed nine predominant chemokine profiles that varied among tumor types, but none of them had ligands for more than one chemokine receptor expressed on mature NK cells.

**Implications of all the available evidence**

We here report a comprehensive study of the chemokine receptor landscape and the migratory behavior of human NK cells. Our results show that chemokine stimulation can elicit a synergistic migratory response in NK cells. We speculate that the sparsity of naturally occurring pairs of chemokines-chemokine receptors may explain the systematic exclusion of NK cells from the tumor microenvironment and represent an untapped potential for engineering next-generation NK-cell based therapies in oncology.

## Introduction

Natural killer (NK) cells are members of the innate lymphoid cell (ILC) family that provide host defense against tumors and pathogen-infected cells.^1^, ^2^, ^3^ Unlike T and B cells, they can recognize and kill target cells without prior sensitization, due to a diverse repertoire of germline-encoded inhibitory and activating receptors on their cell surface.^1^^,^^4^, ^5^, ^6^, ^7^, ^8^, ^9^, ^10^, ^11^ One important mode of target recognition is mediated by the FcγRIII (CD16) triggering antibody-dependent cellular cytotoxicity (ADCC).^12^^,^^13^ NK cells can kill transformed or infected cells by the release of perforin and granzymes or by utilizing effector molecules of the tumor necrosis factor (TNF) family, such as TNF, TNF-related apoptosis-inducing ligand (TRAIL), and Fas ligand (FasL).^14^, ^15^, ^16^ Additionally, upon activation, NK cells rapidly produce chemokines and cytokines, including interferon (IFN)-γ, and GM-CSF, that recruit and affect the function of hematopoietic and non-hematopoietic cells in the tumor microenvironment.^17^, ^18^, ^19^, ^20^ These potent effector functions allow them to play an important role in various diseases, including cancer and infectious diseases.^3^^,^^21^

The repertoire of human NK cells is functionally diversified through a tightly regulated differentiation process characterized by an early transition from CD56^bright^ to CD56^dim^ NK cells, followed by coordinated changes in expression of inhibitory receptors, including NKG2A and killer cell immunoglobulin-like receptors (KIRs).^22^, ^23^, ^24^, ^25^, ^26^, ^27^, ^28^ Along the process, NK cells gradually progress from an immunoregulatory phenotype to highly cytotoxic and mature cells capable of effective immune surveillance of cancer.^22^, ^23^, ^24^^,^^26^^,^^29^^,^^30^ The role of NK cells in cancer is supported by the positive prognostic value of mature NK cell infiltration in a variety of tumors including melanoma, renal cell carcinoma, sarcoma, lung, liver, and breast cancer.^2^^,^^31^, ^32^, ^33^, ^34^ Although NK cell differentiation is a critical determinant of the cytotoxic potential of these cells, little is known about how these events shape the migratory behavior of NK cells.

In order to perform their intended function, NK cells must possess the ability to migrate to different target tissues.^35^, ^36^, ^37^, ^38^, ^39^ This process is mediated by a family of small, secreted cytokines, known as chemokines. By binding to their specific receptor, chemokines guide cellular migration. Trafficking is also facilitated by interactions between adhesion molecules and membrane-bound integrins. Altogether, the expression of all these trafficking molecules can be used as an indicator of cellular localization, activation status, and function of immune effector cells.^35^ The significance of the chemokine-chemokine receptor axis is gaining an increasing recognition in immuno-oncology.^36^, ^37^, ^38^, ^39^ The infiltration of solid tumors by NK cells is relatively low when compared to T cells or macrophages.^34^^,^^40^ Thus, enhancing NK cell trafficking into the TME is one of the main challenges in improving the effectiveness of NK cell therapy against solid tumors.

Here, we have used a combination of high-dimensional flow cytometry, time-of-flight mass cytometry (CyTOF) and single-cell RNA sequencing to map chemokine repertoires and functional migratory profiles in resting and activated primary NK cells as well as induced pluripotent stem cell (iPSC)-derived NK cells (iNK). Our results delineate the natural variation in NK cell chemokine receptor repertoires and provide a guide for engineering the migratory properties of NK cells.

## Methods

### Sample collection and PBMC isolation

Buffy coats from random healthy blood donors were obtained from the Oslo University Hospital Blood bank with written informed consent. The samples were anonymized prior to NK cell isolation. The approval was obtained from the regional committees for medical and health research ethics in Norway: 2018/2485. Peripheral blood mononuclear cells were separated from buffy coats by density gravity centrifugation (Lymphoprep; Axis-Shield) using fretted spin tubes (SepMate; Stemcell Technologies). After isolation, the cells were resuspended in MACS buffer (PBS supplemented with 1% BSA and 2 mM EDTA) and stained or transferred into cell culture medium within 1 h.

### Cell isolation, culture, and stimulation

NK cells were purified from PBMC using negative selection with an AutoMACS Pro Separator (Miltenyi) using NK cell isolation kits (Miltenyi, Cat# 130-092-657). The purity of isolated NK cells was over 90%. A panel of CD56, CD3, CD14, CD19, and a dead cell stain was used for each flow cytometry profiling of stimulated NK cells to ensure both adequate NK cell purity and that the chemokine receptors were profiled exclusively on live NK cells.

Isolated NK cells were cultured in R10 medium (RPMI 1640, 10% fetal bovine serum (FBS), 100 U/ml penicillin, 100 μg/ml streptavidin, 2 mM L-glutamine, 20 mM HEPES, pH 7.0). For experiments with stimulation, 1 or 10 ng/ml of IL-15 (Miltenyi, Cat# 130-095-765) or 100 IU/ml IL-2 (Miltenyi, Cat# 130-097-744) was added to R10 NK cell culture medium. NK cells were stimulated either for 24 h or 7 days. For NK cell expansion experiments, cells were expanded from CD3/CD19-depleted PBMC through co-culture with 200 Gy irradiated K562 feeder cells transfected with a lentiviral construct to express high levels of membrane-bound IL-21 and 4-1BBL, kindly provided by Dr. Dean A. Lee (Nationwide Children's Hospital, Columbus, OH). NK cells were expanded with K562-feeder cells in G-Rex24 (Wilson Wolf) at a 1:2 ratio with a total cell number of 0.5 × 10^6^/cm^2^. Cells were cultured in GMP-grade Stem Cell Growth Medium (SCGM, CellGenix) supplemented with 100 IU/ml human recombinant IL-2 (Proleukin) for 11 days with 60% medium exchange on day 7, and IL-2 replenishment days 4, 7 and 10. The percentage of live CD56^+^ CD3^−^ CD14^−^ CD19^−^ cells after stimulation was over 90% in 24 h experiments and over 80% in longer experiments.

### Derivation and expansion of iNK cells

Human iPSC were first differentiated into hematopoietic progenitors and then into NK cells, as previously described.^41^ The iPSC cell line characterized in this study was previously described in a recent paper by Zhu et al.^41^ Fully differentiated iNK cells were kindly provided to us by Fate Therapeutics.

Briefly, human iPSCs were differentiated into hematopoietic progenitor cells by spin EB formation, in a 10-day process, as previously described.^41^, ^42^, ^43^, ^44^ Then, CD34^+^ cells were subsequently enriched prior to differentiation into iNK cells. At the beginning of the iNK cell differentiation culture, CD34^+^ hematopoietic progenitors were plated on OP9 cells in B0 media containing a 2:1 mixture of Dulbecco modified Eagle medium/Ham F12 (Thermo Fisher Scientific, Cat# 11965092, Cat# 11765054), 2 mM L-glutamine (Thermo Fisher Scientific, Cat# 25030081), 1% penicillin/streptomycin (Thermo Fisher Scientific, Cat# 25030081), 25 μM β-mercaptoethanol (Gibco, Cat# M3148), 10% heat-inactivated human serum AB (Sigma, Cat# H3667-100M), 5 ng/ml sodium selenite (Merck Millipore, Cat# S5261), 50 μM ethanolamine (Sigma, Cat# E0135), 20 mg/ml ascorbic acid (Merck Millipore, Cat# A4544), interleukin-3 (IL-3, R&D, Cat# 203-IL); for first week only), stem cell factor (SCF; R&D, Cat# 7466-SC), interleukin-15 (IL-15; R&D, Cat# 247-ILB), Fml-like tyrosine kinase 3 ligand (FLT3L; R&D, Cat# 207-IL) to support NK cell differentiation from hematopoietic progenitors, as previously described.^41^^,^^45^^,^^46^ The cells were left in these conditions for 20 days receiving weekly media changes until they had developed into CD45^+^CD56^+^CD33^−^CD3^−^ cells as determined by flow cytometry. The percentage of CD45^+^ CD56^+^ CD33^−^ CD3^−^ cells was above 80%. At this point, iNK cells were cryopreserved and kindly provided to us by FATE. We then expanded iNK cells in our laboratory using the irradiated K562 cells with transduced membrane-bound IL-21 and 4-1BBL constructs in supplemented B0 media supplemented with 10 mM Hepes and 100 IU/ml IL-2 for 7 days, in agreement with the ethical approval, REK 2019/333.^41^^,^^47^ K562 cells were propagated in R10 medium (RPMI 1640, 10% fetal bovine serum (FBS), 100 U/ml penicillin, 100 μg/ml streptavidin, 2 mM L-glutamine, 20 mM HEPES, pH 7.0).^47^

### Flow cytometry

Cells were stained for flow cytometric analysis using an appropriate combination of antibodies for 15 min in FACS buffer (phosphate-buffered saline (PBS) pH 7.2, 0.5% bovine serum albumin (BSA), and 2 mM EDTA) in the dark, at room temperature. For intracellular staining with anti-granzyme B-AF700 (clone GB11), cells were fixed and permeabilized using a fixation/permeabilization kit (BD Bioscience Cytofix/Cytoperm) after surface staining. Finally, cells were washed twice. Samples were acquired using BD LSR II or FACSymphony A5 equipped with HTS (BD Biosciences), and the data was analyzed using FlowJo 10.6.1 (BD Biosciences) and CytoExploreR (1.1.0).^48^

The list of used fluorochrome-labelled antibodies can be found in the Supplementary Table S1. All mAbs were titrated and used at dilutions ensuring saturated staining of 1 × 10^6^ cells. Dead cells were labelled using Viobility™ 405/520 Fixable Dye (Miltenyi). Biotin-conjugated antibodies were visualized using streptavidin-Brilliant Violet 605 or 785 (Biolegend). The gates for chemokine receptors and adhesion molecules were set using appropriate FMO-1 controls in each experiment. The chemokine receptor expression score used for linear regression analysis to assess the relationship between chemokine receptor expression and chemokine-induced migratory response was calculated as follows: *log2(MFI of the positive population + 1) ∗ Percentage of Chemokine Receptor Expression*.

### Mass cytometry

PBMCs collected from 20 healthy donors were frozen in 10% DMSO and 90% fetal calf serum and stored in liquid nitrogen. The day before acquisition, PBMCs were thawed, counted, and stained with Cell-ID Intercalator-Rh103 (Fluidigm) for viability testing, followed by Fc blocking reagent and a cocktail of surface antibodies. The list of used antibodies can be found in the Supplementary Table S2. Subsequently, cells were fixed in PBS (without calcium and magnesium) with 2% paraformaldehyde, permeabilized, barcoded using the Cell-ID 20-Plex Barcoding Kit (Fluidigm) and pooled. Samples were then transferred to methanol and stored at −20 °C. On the acquisition day, cells were stained with an intracellular antibody cocktail and labelled with Cell-ID Intercalator-Ir. Samples were supplemented with EQ Four Element Calibration Beads (Fluidigm) and acquired on a CyTOF 2 (Fluidigm) equipped with a SuperSampler (Victorian Airship) at an event rate below 350 events per second. Samples were analyzed in a single run. Antibodies were either obtained pre-labelled from Fluidigm or conjugated with metal isotopes using Maxpar X8 antibody labeling kits (Fluidigm). FCS files were normalized and debarcoded using the R package CATALYST (1.21.1).^49^ Then, the gating and downstream analysis were performed in CytoExploreR (1.1.0).^48^ All CyTOF data was transformed using arcsinh (x/5).

### Chemotaxis assay

To measure NK cell migration, we used Corning® Transwell® plates with inserts (pore size 5.0 μm, Cat# CLS3421). 650 μl R10 medium containing recombinant chemokines manufactured by Peprotech including CCL5 (Cat# 300-06), CCL19 (Cat# 300-29B), CXCL8 (Cat# 200-08M), CXCL10 (Cat# 300-12), CXCL12 (Cat# 300-28A), or CX3CL1 (Cat# 300-31) was placed in the lower chamber of a 24-well Transwell plate (Corning). Each chemokine was titrated to determine the optimal concentration; the range tested was from 0.1 ng/ml to 1000 ng/ml. NK cells (5 × 10^5^) were added in 100 μl R10 medium to the upper chamber (5-μm pore size), and the plates were incubated for 1 h at 37 °C. Then, the cells that migrated to the lower chamber were mixed with flow cytometry counting beads (Invitrogen, Cat# PCB100), stained with antibody cocktail as in section Cell isolation, culture, and stimulation, and acquired. The results are presented as migration index which was calculated as a ratio of cells that have migrated to the bottom well during chemokine-induced migration compared to the cells that have spontaneously migrated to the bottom well, without any chemoattractant present. The migration index was then normalized by counting beads (Invitrogen, Cat# PCB100). The calculations were performed separately for each subset of NK cells.

### Data mining

The single-cell RNA sequencing data from peripheral blood NK cells were obtained from the GEO database, accession number GSE130430.^50^ The count data was then filtered for peripheral blood samples from healthy donors only (2 donors). The bulk gene expression profiles of primary solid tumors were obtained from the combined TCGA, TARGET and GTEx cohort downloaded from the UCSC Xena, filtered for primary solid tumors. The downloaded data was of RSEM tpm type.

### Data analysis

Both single-cell and bulk RNA-seq data were processed using the Seurat (v4.1.0) package in R (v4.1.2).^51^^,^^52^ In the single-cell RNA-seq data, we filtered out the cells that expressed <200 genes or >2500 genes, as well as the cells with >5% mitochondrial transcripts content. Gene expression values for each cell were log normalized and scaled by a factor of 10,000. To avoid biasing the clusters by cell library size or mitochondrial transcript content, gene expression values were scaled based on the number of UMIs in each cell and the cell mitochondrial transcript content. We then combined cells derived from peripheral blood. Naive clustering of the cells into sub-populations was then conducted using Seurat’s implementation of a shared nearest neighbor modularity optimization-based clustering algorithm (Louvain’s original algorithm). Based on the PCElbowPlot, we selected a certain number of principal components (PCs) for the clustering analysis when that number reached the baseline of the standard deviation of PC. Cell clusters were visualized using UMAP. In order to predict cellular differentiation, cells were ordered in pseudotime using monocle3 (v1.2.9) through SeuratWrappers (v0.3.0).^53^^,^^54^ The diversity of chemokine receptor-based clusters in classical clusters was calculated with Shannon diversity index in vegan (v2.6-2) package.^55^ Then, the plots were created using Seurat (v4.1.0), dittoSeq (v1.6.0) and Nebulosa (v1.4.0) packages.^51^^,^^56^, ^57^, ^58^

Bulk RNA-seq data was first filtered for primary solid tumors and loaded into the Seurat. Then, naive clustering of the cells into sub-populations was conducted using Seurat’s implementation of a shared nearest neighbor modularity optimization-based clustering algorithm (Louvain’s original algorithm). Based on the PCElbowPlot, we picked a certain number of principal components (PCs) for the clustering analysis when that number reached the baseline value of the standard deviation of PC. Cell clusters were visualized using UMAP. Then, the graphs were created with Complexheatmap (v.2.10.0) and plotly (v4.10.0) packages.^58^, ^59^, ^60^ Tidyverse packages were used throughout the analysis.^61^^,^^62^

### Statistical methods

Simple linear regression was used to examine the relationship between the migration index and the chemokine receptor expression score. As described above, the migration index was calculated as a ratio of cells that have migrated to the bottom well during chemokine-induced migration compared to the cells that have spontaneously migrated to the bottom well, without any chemoattractant present, normalized by counting beads. The chemokine receptor expression score was calculated as follows: *log2(MFI of the positive population + 1) ∗ Percentage of Chemokine Receptor Expression*. R^2^ was used as a measure of goodness of fit. P values were calculated using Wald’s test.

Differences between the migration index curves from chemokine-induced Transwell migration experiments were assessed using two-way analysis of variance (ANOVA) with post-hoc Dunnett’s test.

### Role of the funding source

The Polish Ministry of Science and Higher Education (DI2018 020548), the National Science Centre, Poland (2020/37/B/NZ6/02191), the Norwegian Cancer Society (grant no 223310), the Research Council of Norway (grant no 275469), the South-Eastern Norway Regional Health Authority (grant no 2021073) and the National Cancer Institute of the National Institutes of Health (Award Number P01CA111412) supported this study. The funders have no role in the study design, the data collection, data analysis, interpretation, and the writing of the report. The corresponding author had full access to all of the data and the final responsibility to submit for publication.

## Results

### Profiling the chemokine receptor repertoire of NK cells by flow cytometry

**Fig. 1:**
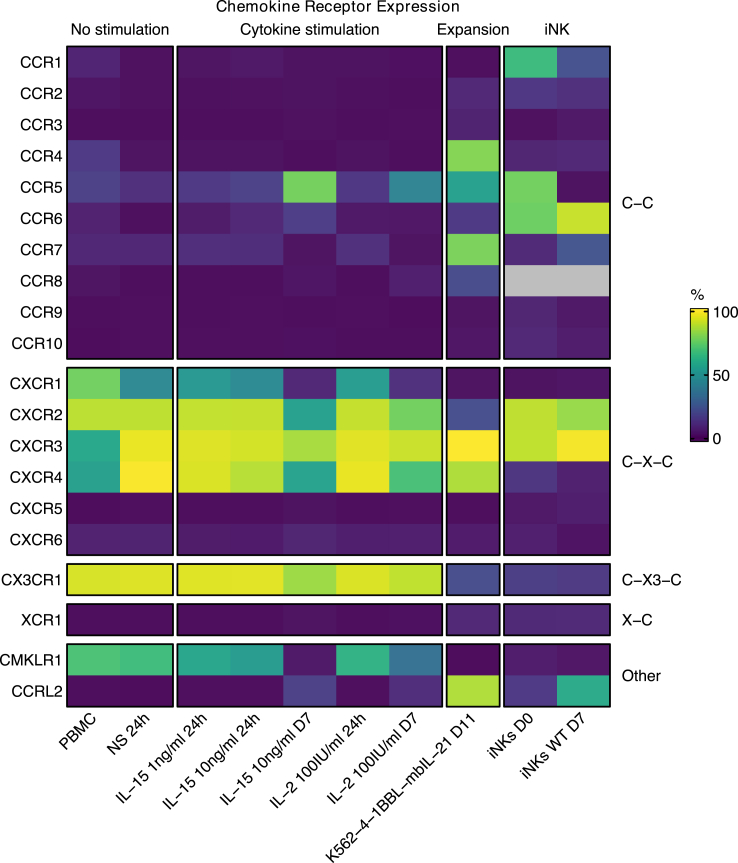
**Chemokine receptor expression in NK cells.** Chemokine receptor expression represented as percentage of cells expressing given chemokine receptor. Missing data is marked with gray. Vertically, chemokine receptors are grouped based on their structure (C–C, CX–C, CX3–C, X–C, and other chemotactic receptors). Horizontally, the NK cells are divided based on the condition (no stimulation, cytokine stimulation, 11-day feeder cell expansion of PBMC-derived NK cells, 7-day expansion of iPSC-derived NK cells). A panel of CD56, CD3, CD14, CD19, and a dead cell stain was used for each flow cytometry profiling to ensure adequate NK cell purity and that the chemokine receptors were profiled exclusively on live NK cells. Each experiment (n = 3) was performed on independent donors in two technical replicates. Data represents means.

To get a comprehensive view of chemokine receptor profile in NK cells, we examined the expression of all classical chemokine receptors and several non-classical chemotactic receptors by flow cytometry in NK cells from PBMC (PB NK), the K562-4-1BBL-mbIL21- or cytokine-expanded NK cells, and iNK cells (Fig. 1). The representative chemokine receptor staining plots as well as the chemokine receptor expression for each donor and each stimuli representing donor-to-donor variability can be found in Supplementary Figures S1 and S2. Flow cytometry analysis of PB NK cells revealed a consistent picture of the chemokine receptor profile, with CXCR1, CXCR2, CXCR3, CXCR4, CX3CR1, and atypical chemotactic receptor CMKLR1, as the most highly expressed (>50%). Additionally, the expression of CCR1, CCR2, CCR4, CCR5, CCR6, CCR7, CCR8 and CXCR6 was also detected, but only on a small fraction of NK cells. A 24-h culture of isolated NK cells without any stimulation slightly altered the profile of chemokine receptors, resulting in the decreased expression of CCR4, CCR6, and CXCR1 and a slight increase in CXCR3 and CXCR4 expression. CCR5, CCR7, CXCR2, CX3CR1 and CMKLR1 receptors showed no significant changes in expression after 24-h culture. We did not detect CCR3, CCR9, CCR10, CXCR5, XCR1 or CCRL2 on PB NK cells.

To explore dynamic changes of the chemokine receptor repertoire, we phenotyped NK cells upon exposure to cytokines (IL-2 or IL-15) associated with the development, differentiation, and acquisition of effector functions by NK cells. Isolated NK cells were cultured and stimulated with two concentrations of IL-15 (1 and 10 ng/ml) or with IL-2 (100 IU/ml) for either 24 h or 7 days. Short-term cytokine stimulation had minimal impact on surface expression of chemokine receptors. However, long-term (7-day) cytokine stimulation resulted in a decreased expression of CXCR1, CXCR2, CX3CR1, CMKLR1, and a marked increase in the expression of CCR5. Although these changes were more pronounced for IL-15 compared to IL-2, stimulation with either of them resulted in the same overarching change in chemokine receptor repertoires (Fig. 1).

We next evaluated series of NK cell-based products with potential clinical application, and we observed a profoundly altered chemokine receptor profile in NK cells subjected to a 11-day expansion protocol using K562-4-1BBL-mbIL21 cells.^47^ Compared to PB NK cells, expanded cells acquired high expression of CCR4, CCR5, CCR7, CCRL2, and especially CXCR3, while losing CXCR1, CXCR2, CX3CR1 and CMKLR1 (Fig. 1). Mature resting and short term expanded iNK cells were characterized by the most unique chemokine receptor signature among the cells tested, with high expression of CXCR2, CXCR3, CCR1, CCR6 and S1P5R, low levels of CXCR1 and CXCR4, and modest expression of CX3CR1 (Fig. 1). Based on this initial screening, we excluded CCR3, CCR9, CCR10, CXCR5, XCR1 or CCRL2 from further experiments.

### Effects of differentiation on chemokine receptor expression and migratory potential of NK cells

We evaluated the expression of selected chemokine receptors on different NK cell subsets in healthy donors with an extended high-dimensional flow cytometry panel. Based on the surface expression of CD56, CD57, NKG2A, NKG2C and four KIRs, NK cells were divided into 6 different subsets, and ordered accordingly to their differentiation status: 1) CD56^bright^, 2) CD56^dim^ NKG2A^+^ CD57^−^ KIR^−^, 3) CD56^dim^ NKG2A^+^ CD57^−^ KIR^+^, 4) CD56^dim^ NKG2A^−^ CD57^−^ KIR^+^, 5) CD56^dim^ NKG2A^−^ CD57^+^ self-KIR^+^, and 6) CD56^dim^ NKG2A^−^ CD57^+^ self-KIR^+^ NKG2C^+^.

**Fig. 2:**
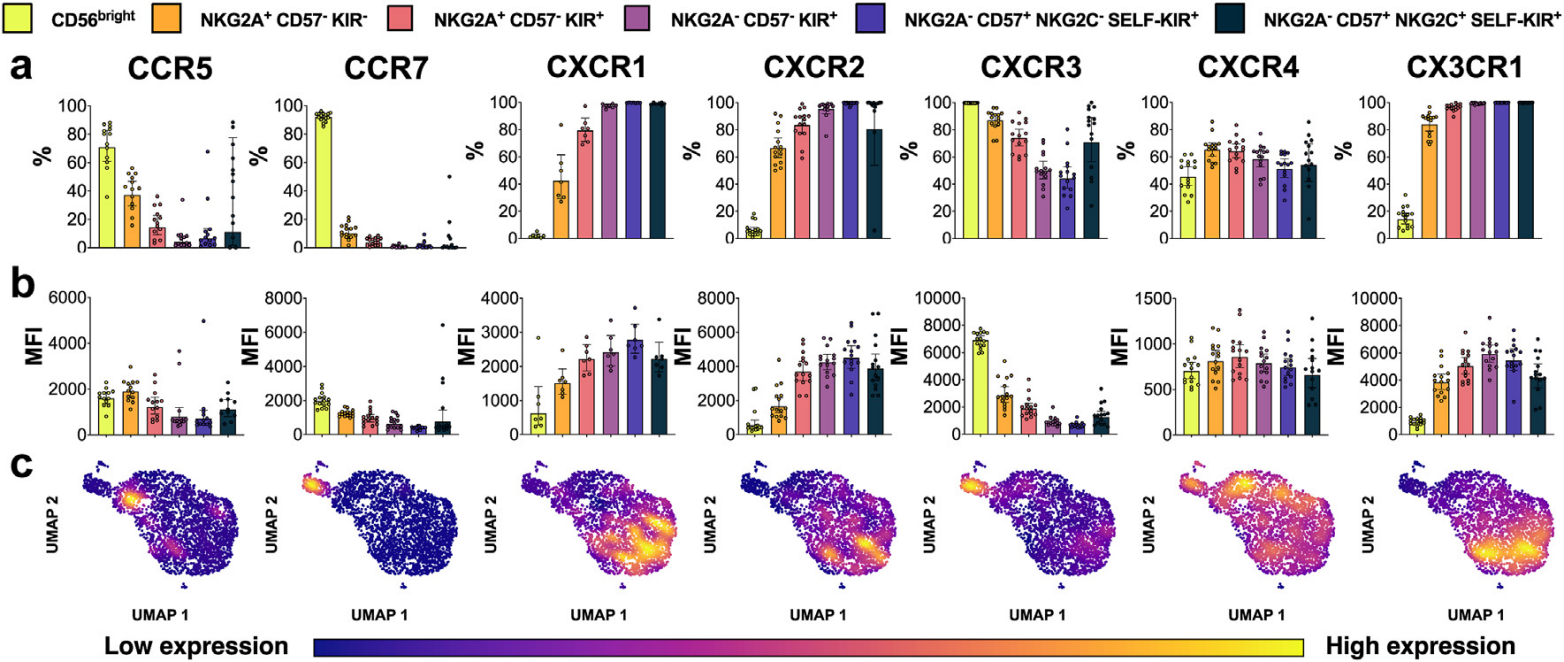
**The effects of differentiation on NK cell chemotactic landscape.** NK cell subsets were identified by CD56, NKG2A, CD57, NKG2C, and KIR expression, and ordered accordingly to their differentiation status. Each heading applies to the whole column. The legend applies to sections (a and b). In (a and b) each dot represents an individual donor. Bars represents geometric mean with 95% confidence interval. Two technical replicates were assessed in each donor. The chemokine receptor expression is presented as (a) percent of cells expressing chemokine receptor, (b) median fluorescent intensity of chemokine receptor in positive population, and (c) chemokine receptor mRNA expression derived from single-cell RNA-seq data. The single-cell RNA-seq clustering and pseudotime analysis is presented in Supplementary Figures S5 and S9. The cells are positioned from the least mature (left, top) to most mature (right, bottom).

We found that the differentiation process was associated with a gradual change in the expression of most of the studied receptors. Both percentage of positive cells and median fluorescent intensity (MFI) of CCR5, CCR7, CCR8 and CXCR3 progressively decreased along with the differentiation (Fig. 2a and b, Supplementary Figure S3). Notably, expression of these chemokine receptors increased slightly in adaptive NK cells compared to the most differentiated but non-adaptive CD56^dim^ NKG2A^−^ CD57^+^ Self-KIR^+^ NKG2C^−^ cells. Conversely, CX3CR1, CXCR1, CXCR2, and CMKLR1 expression levels increased during NK cell differentiation, rising from less than 5% to almost 100% positive cells (Fig. 2a and b, Supplementary Figure S3). Again, the trend was inversed in adaptive NK cells, as their expression levels were slightly lower than in non-adaptive mature NK cells. CCR1, CCR6 and CXCR6 were primarily expressed by NKG2A^+^ positive CD56^dim^ NK cells (Supplementary Figure S3) while CXCR4 expression followed a sinusoidal pattern across all subsets (Fig. 2a and b). Expression of CCR4 was uniform in all subsets, while CCR2 was characterized by low expression, highest in the least mature subsets (Supplementary Figure S3). This data was verified in CyTOF experiment using a panel of chemokine receptors (CCR2, CCR5, CCR7, CXCR1, CXCR2, CXCR3, CXCR4, CX3CR1) in 20 healthy PBMC donors (Supplementary Figure S4).

To confirm the relationship between differentiation and chemokine receptor expression, we analyzed single-cell RNA-seq data from PB NK cells from two healthy donors. First, cells were clustered and ordered accordingly to the differentiation status through trajectory analysis (Supplementary Figure S5). Then, the expression levels of chemokine receptors were projected on UMAP plots. The mRNA expression of all analyzed chemokine receptors showed subset-specific expression patterns that closely matched those observed at the protein level (Fig. 2a–c, Supplementary Figure S3A–S3C).

Next, we analyzed the expression of L-selectin (CD62L), along with other adhesion molecules: P-selectin glycoprotein ligand-1 (Psgl-1, CD162), Integrin B2 (CD18), Integrin B7 chain, ICAM-3 (CD50), and Integrin α M (CD11b) in a similar manner. Likewise, the expression of the adhesion molecules was also modulated by differentiation. The expression of L-selectin and CD44 decreased with differentiation, whereas CD162, CD11b, CD18, and ICAM-3 were upregulated (Supplementary Figure S6).

Based on these experiments, we selected a set of classical chemokine receptors that were expressed on >50% of at least one subpopulation of unstimulated PB NK cells for further characterization and downstream functional experiments. The receptors matching these criteria were CCR5, CCR7, CXCR1, CXCR2, CXCR3, CXCR4, and CX3CR1.

### Effects of differentiation on migratory potential

**Fig. 3:**
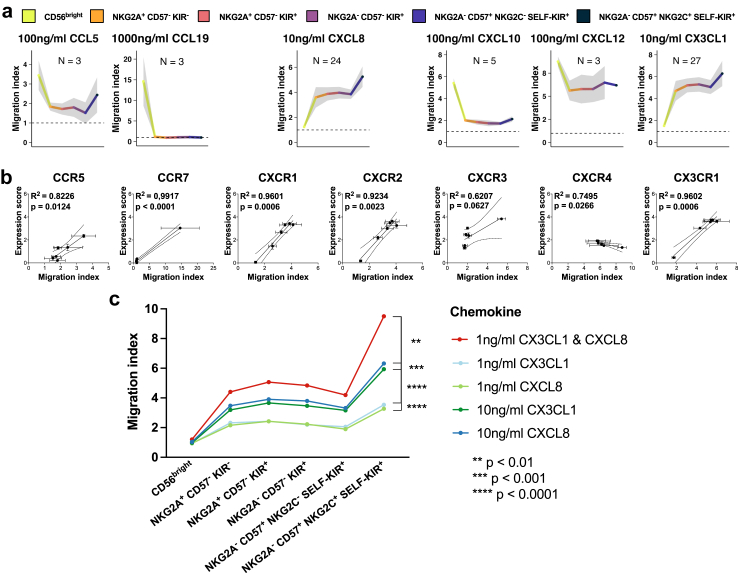
**NK cell chemokine-induced migratory responses in subset resolution.** NK cell subsets were identified by CD56, NKG2A, CD57, NKG2C, and KIR expression, and ordered accordingly to their differentiation status. (a) Transwell analysis of chemokine induced migration in a subset resolution. Migration index was calculated as a ratio of cells that have migrated to the bottom well during chemokine-induced migration compared to the cells that have spontaneously migrated to the bottom well, without any chemoattractant present. Data represents means ± SEM (gray area). Some chemokines (CX3CL1, CXCL8) were evaluated in more donors as they were used for the further experiments evaluating potential synergies (c). (b) A linear regression analysis between migration index and respective chemokine receptor protein expression (Fig. 2a and b). The chemokine receptor expression score was calculated as follows: *log2(MFI of the positive population + 1) ∗ Percentage of Chemokine Receptor Expression*. P values were calculated by Wald’s test. (c) Transwell analysis of combined effects between two chemokines in subset resolution. The experiment was performed on 10 donors. Data represents geometric mean. The differences between the curves were assessed using two-way ANOVA with post-hoc Dunnett’s test.

To determine whether the observed differences in chemokine receptor expression between distinct subsets of NK cells affect their migratory potential, we employed the Transwell system. Solely the ligands of previously selected chemokine receptors (CCR5, CCR7, CXCR1, CXCR2, CXCR3, CXCR4, and CX3CR1) were included in these functional experiments. CX3CL1- and CXCL8-induced migration capability correlated with the differentiation status of NK cells, as did the expression pattern of their corresponding receptors (Fig. 3a). Conversely, CCL19, CCL5, and CXCL10-induced migration capability decreased with NK cell differentiation, which corresponded to a decrease in receptor expression (Fig. 3a). In general, we observed that the chemokine-induced migration capability of each subset of NK cells reflected the expression level of the corresponding chemokine receptor. To assess the relationship between chemokine receptor protein levels and functional migration, we performed linear regression analysis which showed very high coefficients of determination (R^2^) for nearly all chemokines tested (CCL5, CCL19, CXCL8, CXCL12, and CX3CL1) with R^2^ ranging from 0.7495 to 0.9917, indicating that the surface expression of chemokine receptor is a critical factor determining migratory response to its ligand (Fig. 3b). We found that the optimal formula representing chemokine receptor expression, closely correlating with migratory response was *log2(MFI of the positive population + 1) ∗ Percentage of Chemokine Receptor Expression*. In this context, adaptive NKG2C^+^ NK cells stood out from other subsets, as they had a unique chemokine repertoire and migratory profile, with higher intrinsic migratory potential despite lower expression of chemokine receptors such as CXCR1, CXCR2, CXCR4, and CX3CR1, when compared to non-adaptive mature NK cells. We also found CXCR4 expression to be inversely correlated with CXCL12-induced migration due to high CXCL12-induced migratory response and relatively low CXCR4 expression in CD56^bright^ NK cells. This is likely due to higher intrinsic migratory potential of CD56^bright^ NK cells and is in agreement with the literature reporting accumulation of CD56^bright^ NK cells in tissues with high CXCL12 expression.^63^, ^64^, ^65^

Our flow cytometry data indicated that some NK cell subsets express multiple chemokine receptors. Therefore, we examined whether simultaneous ligation of multiple chemokine receptors would provide synergistic effects on NK cell migration. After titration experiments performed to find the concentrations needed to achieve peak migratory responses (Supplementary Figure S7), we stimulated the NK cells with optimal (10 ng/ml) or suboptimal (1 ng/ml) concentrations of CX3CL1 or CXCL8, as well as suboptimal concentration of both chemokines simultaneously (1 ng/ml CX3CL1 with 1 ng/ml CXCL8). Within all CD56^dim^ subsets of NK cells, the combination of both chemokines induced a stronger migratory response than both suboptimal and optimal concentrations of each chemokine, confirming the presence of a synergistic effect between chemokine receptor stimulation in NKG2C^+^ CD57^+^ adaptive NK cells and additive effect in other CD56^dim^ NK cell subsets, emphasizing the importance of studying the entire chemokine-chemokine receptor landscape (Fig. 3c, Supplementary Figure S8).

### Exploring chemokine receptor co-expression patterns

**Fig. 4:**
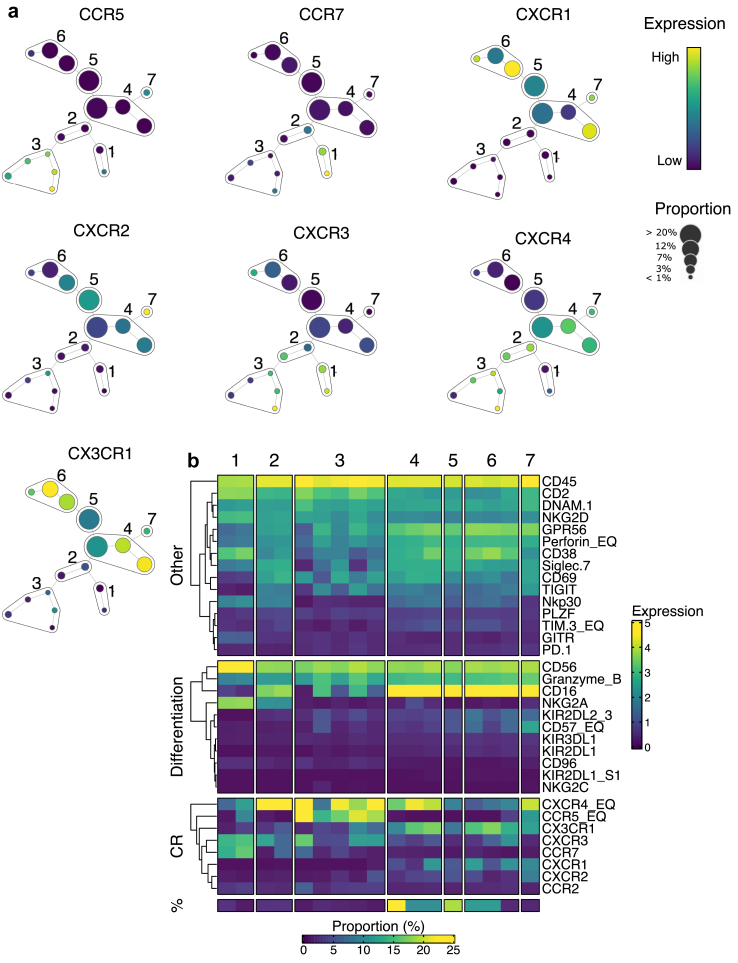
**Mass cytometry profiling of chemokine receptor co-expression on peripheral blood NK cells.** (a) Results of SPADE clustering of peripheral blood NK cells based on expression of 7 chemokine receptors: CCR5, CCR7, CXCR1, CXCR2, CXCR3, CXCR4, CX3CR1. The size of the circles reflects number of cells in the group. The color scale reflects the chemokine receptor expression. (b) Expression levels (MFI) of chemokine receptors, NK cell differentiation markers and other NK cell receptors in SPADE clusters (1–7). The bottom row of the heatmap shows the frequency of each cluster in bulk peripheral blood NK cells. The experiment was performed on samples from 20 healthy donors.

Discovering synergistic effects of simultaneous chemokine receptor ligation prompted us to conduct a more detailed mapping of chemokine diversity within the NK cell repertoire. We designed a mass cytometry (CyTOF) panel to perform a parallel assessment of CCR5, CCR7, CXCR1, CXCR2, CXCR3, CXCR4, and CX3CR1 in the NK cell compartment. This chemokine-centric approach revealed that co-expression patterns of chemokine receptors alone could accurately identify classical NK cell subsets, such as CD56^bright^, based on high CXCR3 and CCR7 expression, and CD56^dim^ NKG2A^+^ based on high expression of CXCR4 and modest expression of CXCR3 (Fig. 4). Interestingly, in more differentiated NK cell subsets (4,5,6,7) the expression patterns of chemokine receptors showed increasing complexity, suggesting an additional layer of functional diversification in subsets with identical expression of NK cell receptors and canonical markers (Fig. 4).

To corroborate our observation of increased chemokine receptor diversity in more differentiated NK cell subsets, we re-clustered single-cell RNA-seq data based on the expression of CCR5, CCR7, CXCR1, CXCR2, CXCR3, CXCR4, and CX3CR1. We identified 8 clusters with unique chemokine receptor combinations (Supplementary Figure S9A). These clusters were plotted onto the previously created UMAP plot (Supplementary Figure S5A and S9B) with a known differentiation trajectory (Supplementary Figure S9C). We noticed a substantial heterogeneity of chemokine receptors in NK cells, which was progressively increasing along with differentiation. This observation was further verified by the Shannon diversity index calculated for each of the “classical” clusters associated with NK differentiation (Supplementary Figure S9D). Our data indicates that NK cell differentiation not only alters chemokine receptor profile in a coordinated fashion but also does it in a way that progressively increases the overall diversity of chemokine receptors in NK cells.

### Chemokine-based clustering of malignant tumors

**Fig. 5:**
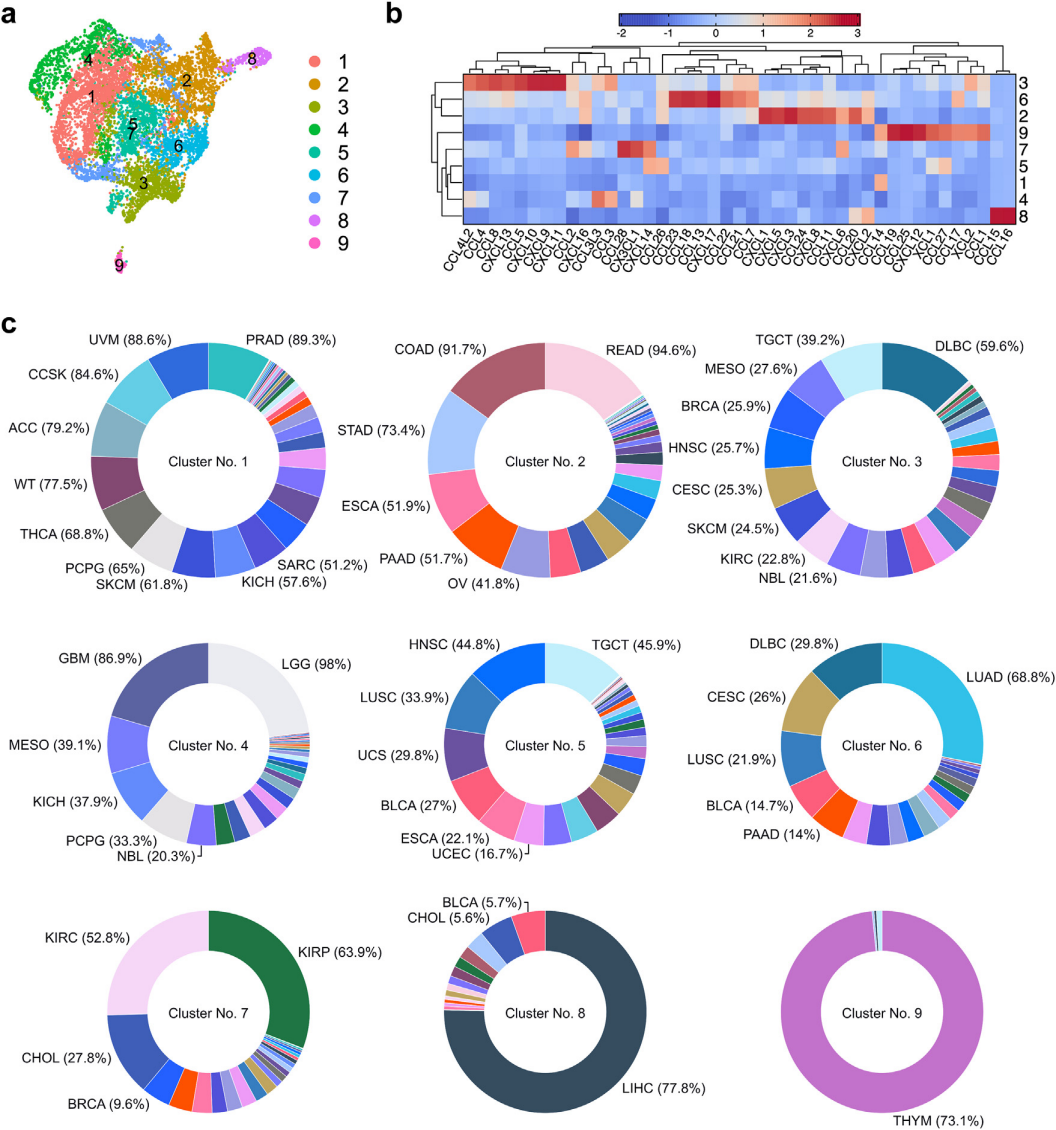
**Chemokine landscape of 9471 primary solid tumors from TCGA and TARGET cohorts.** (a) UMAP plot visualizing the results of clustering 9471 primary solid tumors from TCGA and TARGET cohorts based on chemokine expression. (b) Expression of chemokines across nine identified clusters. (c) Proportion of cluster assignment for each tumor visualized as donut plots. ACC, adrenocortical carcinoma; BLCA, bladder urothelial carcinoma; LGG, brain lower grade glioma; BRCA, breast invasive carcinoma; CESC, cervical squamous cell carcinoma and endocervical adenocarcinoma; CHOL, cholangiocarcinoma; CCSK, clear cell sarcoma of the kidney; COAD, colon adenocarcinoma; DLBC, diffuse large B-cell lymphoma; ESCA, esophageal carcinoma; GBM, glioblastoma multiforme; HNSC, head and neck squamous cell carcinoma; KICH, kidney chromophobe; KIRC, kidney renal clear cell carcinoma; KIRP, kidney renal papillary cell carcinoma; LIHC, liver hepatocellular carcinoma; LUAD, lung adenocarcinoma; LUSC, lung squamous cell carcinoma; MESO, mesothelioma; NBL, neuroblastoma; OV, Ovarian serous cystadenocarcinoma; PAAD, pancreatic adenocarcinoma; PCPG, pheochromocytoma and paraganglioma; PRAD, prostate adenocarcinoma; READ, rectum adenocarcinoma; SARC, sarcoma; SKCM, skin cutaneous melanoma; STAD, stomach adenocarcinoma; TGCT, testicular germ cell tumors; THYM, thymoma; THCA, thyroid carcinoma; UCS, uterine carcinosarcoma; UCEC, uterine corpus endometrial carcinoma; UVM, uveal melanoma; WT, wilms tumor.

The detailed profiling and functional assessment of primary PB NK cells in terms of chemokine receptor landscape suggested an increasing complexity associated with NK cell differentiation, along with a broad overlap in the expression of chemokine receptors across distinct subsets. We next addressed whether and how the underlying diversity in the chemokine repertoire could explain the sparsity of NK cell in solid tumors or identify suitable targets for NK cell therapy. To this end, we analyzed the chemokine landscape in solid tumors by mining RNA-seq data from TCGA and TARGET databases. Using harmonized RNA-seq data from TCGA and TARGET cohorts, we clustered 9471 primary solid tumors based on chemokine expression. Louvain algorithm-based clustering implemented in Seurat revealed 9 distinct patterns of chemokine co-expression within the tumors (Fig. 5a and b). Further, to assess the composition of each cluster, we calculated the proportion of cluster assignment for each tumor (Fig. 5c). The results showed that some clusters e.g., cluster 7 or 8, comprised a single histological tumor type (hepatocellular carcinoma (LIHC) and thymoma (THYM), respectively), whereas clusters 2 and 4 represented a significant fraction of more than 10 different histological tumor types. Strikingly, none of the clusters showed expression of ligands for more than one chemokine receptor on mature NK cells. The chemokine expression profile of each cluster allowed us to identify cluster 3 as the one most favoring the infiltration of expanded NK cells and potentially enhancing the efficacy of NK cell therapy, because of high CXCR3 and CCR4 expression on expanded NK cells. Since nearly 60% of DLBCL tumors have a chemokine profile of cluster 3, this tumor would appear to be a suitable target for evaluation of unmodified expanded NK cell therapy.

## Discussion

NK cells are important innate effector cells in the human immune system.^3^ Their function far exceeds the role initially attributed to them as a sentinel patrolling the human body. They are a group of cells residing in both lymphoid and non-lymphoid tissues and performing additional functions such as participating in the normal development of spiral vessels during pregnancy or modulating T-cell polarization through the secretion of cytokines.^3^^,^^66^, ^67^, ^68^, ^69^ The multifaceted role of NK cells and the high phenotypic and functional diversity of this population requires adequate trafficking capability, which is controlled by a specific repertoire of chemotactic receptors, responding to environmental signals mediated by chemokines.^70^ While the effects of differentiation on NK cell cytotoxicity, cytokine secretion, proliferation and metabolism are well documented, previous studies provide limited and fragmented information regarding the expression of chemokine receptors among different NK cell subsets, without concluding whether it is regulated by mechanisms related to the functional maturation of these cells and the stimulatory effects of cytokines.^22^^,^^69^^,^^71^ Using high-dimensional flow and mass cytometry, we corroborate and extend previous studies, showing that NK cells express a wide range of chemotactic receptors, identifying CCR5, CCR7, CXCR1, CXCR2, CXCR3, CXCR4 and CX3CR1 receptors among those most abundantly expressed on the surface of NK cells.^72^^,^^73^ Importantly, multi-parameter profiling of PB NK cells by mass cytometry revealed distinct and coordinated chemokine repertoires associated with NK cell differentiation process.

Each chemokine has its own specific set of functions in the immune system, based on which it can be classified as mostly related to innate immunity, adaptive immunity, or both.^74^ In this study, we found that receptors associated with adaptive immunity, characteristic for T cells, such as CCR5, CCR7 and CXCR3, were most prominently expressed in immature CD56^bright^ NK cells, while receptors associated with innate immunity (neutrophils, macrophages) were expressed in mature NK cells. This implies vast differences in functionality of NK cell subsets and supports the notion that immature CD56^bright^ NK cells are not a dysfunctional subset being solely the precursor to mature NK cell subsets, rather they are an important self-standing component of the human immune system. CD56^bright^ NK cells are considered efficient cytokine producers with immunoregulatory properties.^69^ In this context, our data indicates that the chemokine receptor profile of CD56^bright^ NK cells is essential for their function, by facilitating their migration to lymph nodes where upon endogenous T cell-derived IL-2 or dendritic cell-derived IL-12, CD56^bright^ NK cells secrete T helper type 1 (Th1) cytokines, such as IFN-γ and TNF-α, that favor Th1 T cell polarization and influence the developing antigen-specific immune response.^69^^,^^75^^,^^76^

Mature NK cells are on the other side of the functionality spectrum. While under certain conditions they can also be potent producers of cytokines, their main proposed function is to directly kill virus-infected cells and tumor cells through cell-mediated cytotoxicity.^71^ Here, we demonstrate that their chemokine receptor repertoire (CXCR1/2^+^, CX3CR1^+^) is similar to that of patrolling monocytes and neutrophils, indicating that their role is likely to involve patrolling peripheral tissues.^77^^,^^78^ The repertoire of neutrophil-like chemokine receptors may also be important for neutrophil-NK cell crosstalk, which has recently been shown to be an important process in NK cell maturation.^79^

As chemokine receptors cooperate with adhesion molecules such as selectins and integrins to guide cell migration, we analyzed the expression of L-selectin (CD62L), P-selectin glycoprotein ligand-1 (Psgl-1, CD162), Integrin B2 (CD18), Integrin B7 chain, ICAM-3 (CD50), and Integrin α M (CD11b) in NK cell subsets. Similarly to chemokine receptors, we found that their levels were modulated by differentiation as the expression of L-selectin and CD44 decreased with NK cell differentiation, while CD162, CD11b, CD18, and ICAM-3 were upregulated. The decline in CD62L in more mature NK cells was previously reported.^80^ Notably, functionally related chemokine receptors and adhesion molecules, e.g., CD62L and CCR7 (secondary lymphoid organ homing via HEV), CX3CR1-CD162 (extravasation), followed the same expression pattern in subsets at different stages of NK cell differentiation.

The profile of chemokine receptors among NK cells, although relatively stable, is not fixed and can change dynamically. In this study, we found that external stimuli affect its composition both in the short and long timeframe. Culture of isolated PB NKs without additional stimulation can alter the quantitative expression of the major receptors, leading to a decrease in CXCR1 and CXCR2 with an increase in CXCR3 and CXCR4 expression. Stimulation with both IL-2 and IL-15 in long-term cultures (7-day) resulted in a complete loss of CXCR1 expression, downregulation of CXCR2, CXCR4, CX3CR1 and CMKLR1 with a marked upregulation of CCR5 expression. The plasticity and responsiveness of NK cells to environmental stimuli, as reflected by profound changes in profile of chemokine receptors, is also indicated by the results of NK cells undergoing expansion with K562-based feeder cells with membrane-bound IL-21 and 4-1BBL, which shifts NK cells toward responsiveness to chemokines associated with adaptive immunity. Since physiological concentrations of cytokines modulate chemokine receptor expression and therefore affect NK cell migration, they may be used to enhance the efficacy of cell therapies. However, our study also highlights the risk for diverting their homing away from the intended target organ.

To assess whether differences in the expression of surface chemokine receptors correspond to the chemokine responsiveness of different subsets of NK cells, we used the Transwell system coupled with high-dimensional flow cytometry. We observed that the migratory response was closely associated with the expression of the corresponding chemokine receptor, indicating that it is the most important factor determining chemokine responsiveness. Furthermore, we investigated whether simultaneous ligation of multiple chemokine receptors would provide synergistic effects allowing to bypass the limitations of a single ligand-receptor chemokine pair. To this end, we stimulated NK cells with optimal and suboptimal concentrations of CXCL8 and CX3CL1, as well as a combination of suboptimal concentrations of both chemokines. We found that simultaneous ligation with suboptimal concentrations of both chemokines resulted in a stronger migratory response than both suboptimal and optimal concentration of each chemokine separately. Thus, equipping NK cells for adoptive therapy with more than one chemokine receptor to guide NK cells can enhance tumor infiltration compared to the use of a single chemokine receptor. Such an approach would also reduce the chance of tumor immune evasion through loss of expression of one of the targeted chemokines.

The observation of synergistic effects following simultaneous chemokine receptor ligation prompted us to carry out a more detailed mapping of chemokine receptor co-expression using mass cytometry. Clustering of PB NK cells based on chemokine receptor expression revealed that co-expression patterns alone could accurately identify immature classical NK cell subsets, such as CD56^bright^ and CD56^dim^ NKG2A^+^ cells. However, in differentiated NK cell subsets, the expression patterns of chemokine receptors were more diverse. This finding adds another layer to the functional diversification of NK cells based on migratory potential. In this context, the most mature adaptive NKG2C^+^ cells showed a unique chemokine receptor profile, with high expression of receptors typical of both mature and immature NK cells. Additionally, despite lower expression of CXCR1, CXCR2, CXCR4 and CX3CR1 than CD56^dim^ CD57^+^ NKG2A^−^ KIR^+^ NK cells, their migratory response was more potent. This indicates that other important factors, besides chemokine receptor expression, come into play and influence the strength of chemokine-induced NK cell migration.

The ability of cells to infiltrate a tumor is a fundamental prerequisite for anti-tumor immunity.^35^^,^^39^^,^^81^ The chemokine receptor co-expression patterns and their synergistic action, prompted us to search for potential reasons explaining the low abundance of mature NK cells in solid tumors, as well as for appropriate tumor types that could be effectively targeted by adoptive NK cell therapy.^32^^,^^35^ In the context of chemokine receptor synergy following simultaneous ligation, we classified primary solid tumors by re-clustering 9471 primary solid tumors (35 different tumor types) from the TCGA and TARGET cohorts based solely on chemokine expression. We identified nine clusters with distinct chemokine expression patterns. Strikingly, none of them expressed chemokine ligands for more than one chemokine receptor expressed on mature NK cells (CXCR1/2, CXCR4, CX3CR1). On the other hand, the chemokine receptor profile (CCR5^+^, CCR7^+^, CXCR3^+^) of Th1 T cells allows for dual chemokine receptor ligation in clusters 3, 6, and 9.^82^, ^83^, ^84^, ^85^, ^86^ Myeloid cells are even more compatible with the chemokine milieu of solid tumors, with four clusters (3, 4, 6, 7) ligating more than one chemokine receptor expressed on classical monocytes (CCR1, CCR2, CX3CR1).^87^, ^88^, ^89^ Thus, the selection pressure during early stages of tumor microevolution appears to favor infiltration of immunosuppressive cells while excluding mature NK cells with antitumor potential, possibly through avoidance of NK cell chemokine receptor synergy. Importantly, we consider CXCR1 and CXCR2 as one as they are naturally co-expressed in immune cells and share CXCL8 as their main ligand.^90^ We speculate that the lack of synergistic chemokine input may be one factor contributing to the sparse infiltration of mature NK cells in solid tumors. Further work is needed to explore how these distinct chemokine networks correlate with NK cell infiltration in more complex *in vitro* and *in vivo* models and ultimately how this relate to clinical outcomes.

Two of the discovered clusters of primary tumors (cluster 3 and 6) showed expression of multiple ligands for chemokine receptors on expanded NK cells. Cluster 3 was characterized by high expression of CCL3, CCL4, CCL4L2, CCL5 (CCR5 ligands), CXCL9, CXCL10, CXCL11 (CXCR3 ligands) and moderate expression of CCL21 (CCR7 ligand) and CCL22 (CCR4 ligand), while cluster 6 expressed high levels of CCL21 (CCR7 ligand) and CCL22 (CCR4 ligand). Together, almost 90% of DLBCL tumors belong to one of these clusters, indicating that diffuse large B-cell lymphoma may be a permissive target for adoptive feeder cell-based NK cell therapy in terms of its chemokine milieu. As the role of infiltrating NK cells in DLBCL is already partially established as high circulating NK cell count is associated with improved outcomes, it further reinforces the idea of NK cell-based immunotherapy.^91^^,^^92^ Aside from the importance of natural cytotoxicity, NK cell-mediated ADCC plays a crucial role in improving treatment outcomes of patients treated with rituximab using R-CHOP regimen.^91^^,^^92^

Another promising therapeutic approach is iPSC-derived NK cells. iNKs can be fine-tuned through numerous genetic modifications to achieve stronger effector function, introduce tailored specificities and promote persistence, and are therefore attractive candidates for cancer immunotherapy.^93^ Profiling of the chemokine receptor repertoire on one example of a multi-edited iNK line showed that the chemokine receptor profile shared similarities with PB NK cells, e.g., high CXCR2 and CXCR3 expression, but also had some unique features e.g., high expression of CCR1 and CCR6. These data need to be interpreted with some caution given that different iNK cell clones may vary in their receptor repertoire. Given the unique possibility to perform multiple gene edits in the iNK cell platform, adding one or more homing receptors to promote synergistic signaling and homing to the tumor type seems like an attractive approach to enhance iNK-cell based immunotherapy.

In summary, our data provide a comprehensive map of the chemokine receptor landscape in NK cells and reveal an unexpected diversity in the chemokine receptor co-expression that collectively control migratory responses to chemokine gradients. Our observations may also serve as a basis for engineering next-generation adoptive NK cell therapies against hematological malignancies and solid tumors.

## Contributors

Conceptualization–M.L., K.-J.M., data curation–M.L., K.-J.M., formal analysis–M.L., K.-J.M., funding acquisition–M.L., K.-J.M., R.Z., investigation–M.L., K.Z., D.P., M.K., H.J.H., M.T.W., methodology–M.L., M.K., H.J.H., M.T.W., project administration–M.L., L.K., R.Z., K.-J.M., resources–M.L., K.Z., W.H., R.Z., K.-J.M., software–M.L., supervision–R.Z., K.-J.M., validation–L.P., L.K., verification of the underlying data–M.L., K.-J.M., visualization–M.L., K.-J.M., writing—original draft–M.L., K.-J.M., and writing—review & editing–M.L., K.Z., W.H., R.Z., K.-J.M. All authors have read and approved the final version of the manuscript.

## Data sharing statement

The datasets used and/or analyzed during the current study available from the corresponding authors on reasonable request.

## Declaration of interests

Karl-Johan Malmberg is a consultant and has research grants from Fate Therapeutics Inc., and is a member of the advisory board at Vycellix. Radoslaw Zagozdzon is an ad hoc scientific consultant for Pure Biologics S.A. (Wroclaw, Poland) and 4Cell Therapies S.A. (Gliwice, Poland). The remaining authors declare no conflict of interest.

## References

[1] Kiessling R., Klein E., Pross H., Wigzell H. (1975). "Natural" killer cells in the mouse. II. Cytotoxic cells with specificity for mouse Moloney leukemia cells. Characteristics of the killer cell. Eur J Immunol.

[2] Malmberg K.-J., Carlsten M., Björklund A., Sohlberg E., Bryceson Y.T., Ljunggren H.-G. (2017). Natural killer cell-mediated immunosurveillance of human cancer. Semin Immunol.

[3] Vivier E., Tomasello E., Baratin M., Walzer T., Ugolini S. (2008). Functions of natural killer cells. Nat Immunol.

[4] Bauer S., Groh V., Wu J. (1999). Activation of NK cells and T cells by NKG2D, a receptor for stress-inducible MICA. Science.

[5] Sivori S., Parolini S., Falco M. (2000). 2B4 functions as a co-receptor in human NK cell activation. Eur J Immunol.

[6] Bottino C., Castriconi R., Pende D. (2003). Identification of PVR (CD155) and Nectin-2 (CD112) as cell surface ligands for the human DNAM-1 (CD226) activating molecule. J Exp Med.

[7] Lanier L.L. (2008). Up on the tightrope: natural killer cell activation and inhibition. Nat Immunol.

[8] Moretta A., Bottino C., Vitale M. (1996). Receptors for HLA class-I molecules in human natural killer cells. Annu Rev Immunol.

[9] Sivori S., Vitale M., Morelli L. (1997). p46, a novel natural killer cell-specific surface molecule that mediates cell activation. J Exp Med.

[10] Vitale M., Bottino C., Sivori S. (1998). NKp44, a novel triggering surface molecule specifically expressed by activated natural killer cells, is involved in non-major histocompatibility complex-restricted tumor cell lysis. J Exp Med.

[11] Pende D., Parolini S., Pessino A. (1999). Identification and molecular characterization of NKp30, a novel triggering receptor involved in natural cytotoxicity mediated by human natural killer cells. J Exp Med.

[12] Dall’Ozzo S.B., Tartas S., Paintaud G. (2004). Rituximab-dependent cytotoxicity by natural killer cells: influence of FCGR3A polymorphism on the concentration-effect relationship. Cancer Res.

[13] Herberman R.B., Bartram S., Haskill J.S., Nunn M., Holden H.T., West W.H. (1977). Fc receptors on mouse effector cells mediating natural cytotoxicity against tumor cells. J Immunol.

[14] Prager I., Liesche C., Van Ooijen H. (2019). NK cells switch from granzyme B to death receptor–mediated cytotoxicity during serial killing. J Exp Med.

[15] Arase H., Arase N., Saito T. (1995). Fas-mediated cytotoxicity by freshly isolated natural killer cells. J Exp Med.

[16] Zamai L., Ahmad M., Bennett I.M., Azzoni L., Alnemri E.S., Perussia B. (1998). Natural killer (NK) cell–mediated cytotoxicity: differential use of TRAIL and Fas ligand by Immature and mature primary human NK cells. J Exp Med.

[17] Fauriat C., Long E.O., Ljunggren H.-G., Bryceson Y.T. (2010). Regulation of human NK-cell cytokine and chemokine production by target cell recognition. Blood.

[18] Morandi B., Bougras G., Muller W.A., Ferlazzo G., Münz C. (2006). NK cells of human secondary lymphoid tissues enhance T cell polarization via IFN-γ secretion. Eur J Immunol.

[19] Bluman E.M., Bartynski K.J., Avalos B.R., Caligiuri M.A. (1996). Human natural killer cells produce abundant macrophage inflammatory protein-1 alpha in response to monocyte-derived cytokines. J Clin Invest.

[20] Fehniger T.A., Shah M.H., Turner M.J. (1999). Differential cytokine and chemokine gene expression by human NK cells following activation with IL-18 or IL-15 in combination with IL-12: implications for the innate immune response. J Immunol.

[21] Mujal A.M., Delconte R.B., Sun J.C. (2021). Natural killer cells: from innate to adaptive features. Annu Rev Immunol.

[22] Björkström N.K., Riese P., Heuts F. (2010). Expression patterns of NKG2A, KIR, and CD57 define a process of CD56^dim^ NK-cell differentiation uncoupled from NK-cell education. Blood.

[23] Pfefferle A., Jacobs B., Haroun-Izquierdo A., Kveberg L., Sohlberg E., Malmberg K.-J. (2020). Deciphering natural killer cell homeostasis. Front Immunol.

[24] Jacobs R., Stoll M., Stratmann G., Leo R., Link H., Schmidt R. (1992). CD16^−^ CD56^+^ natural killer cells after bone marrow transplantation. Blood.

[25] Freud A.G., Yokohama A., Becknell B. (2006). Evidence for discrete stages of human natural killer cell differentiation in vivo. J Exp Med.

[26] Romagnani C., Juelke K., Falco M. (2007). CD56^bright^CD16^−^ killer Ig-like receptor^−^ NK cells display longer telomeres and acquire features of CD56^dim^ NK cells upon activation. J Immunol.

[27] Béziat V., Descours B., Parizot C., Debré P., Vieillard V. (2010). NK cell terminal differentiation: correlated stepwise decrease of NKG2A and acquisition of KIRs. PLoS One.

[28] Gineau L., Cognet C., Kara N. (2012). Partial MCM4 deficiency in patients with growth retardation, adrenal insufficiency, and natural killer cell deficiency. J Clin Invest.

[29] Nolibe D., Poupon M.F. (1986). Enhancement of pulmonary metastases induced by decreased lung natural killer cell activity. J Natl Cancer Inst.

[30] Crowe N.Y., Smyth M.J., Godfrey D.I. (2002). A critical role for natural killer T cells in immunosurveillance of methylcholanthrene-induced sarcomas. J Exp Med.

[31] Kärre K., Ljunggren H.G., Piontek G., Kiessling R. (1986). Selective rejection of H-2-deficient lymphoma variants suggests alternative immune defence strategy. Nature.

[32] Nersesian S., Schwartz S.L., Grantham S.R. (2021). NK cell infiltration is associated with improved overall survival in solid cancers: a systematic review and meta-analysis. Transl Oncol.

[33] Lachota M., Vincenti M., Winiarska M., Boye K., Zagożdżon R., Malmberg K.J. (2020). Prospects for NK cell therapy of sarcoma. Cancers.

[34] Remark R., Alifano M., Cremer I. (2013). Characteristics and clinical impacts of the immune environments in colorectal and renal cell carcinoma lung metastases: influence of tumor origin. Clin Cancer Res.

[35] Ran G.H., Lin Y.Q., Tian L. (2022). Natural killer cell homing and trafficking in tissues and tumors: from biology to application. Signal Transduct Target Ther.

[36] Domagala J., Lachota M., Klopotowska M. (2020). The tumor microenvironment-A metabolic obstacle to NK cells' activity. Cancers.

[37] Habif G., Crinier A., André P., Vivier E., Narni-Mancinelli E. (2019). Targeting natural killer cells in solid tumors. Cell Mol Immunol.

[38] Tong L., Jiménez-Cortegana C., Tay A.H.M., Wickström S., Galluzzi L., Lundqvist A. (2022). NK cells and solid tumors: therapeutic potential and persisting obstacles. Mol Cancer.

[39] Melero I., Rouzaut A., Motz G.T., Coukos G. (2014). T-cell and NK-cell infiltration into solid tumors: a key limiting factor for efficacious cancer immunotherapy. Cancer Discov.

[40] Gauthier L., Morel A., Anceriz N. (2019). Multifunctional natural killer cell engagers targeting NKp46 trigger protective tumor immunity. Cell.

[41] Zhu H., Blum R.H., Bjordahl R. (2020). Pluripotent stem cell–derived NK cells with high-affinity noncleavable CD16a mediate improved antitumor activity. Blood.

[42] Valamehr B., Abujarour R., Robinson M. (2012). A novel platform to enable the high-throughput derivation and characterization of feeder-free human iPSCs. Sci Rep.

[43] Valamehr B., Robinson M., Abujarour R. (2014). Platform for induction and maintenance of transgene-free hiPSCs resembling ground state pluripotent stem cells. Stem Cell Rep.

[44] Tsutsui H., Valamehr B., Hindoyan A. (2011). An optimized small molecule inhibitor cocktail supports long-term maintenance of human embryonic stem cells. Nat Commun.

[45] Hermanson D.L., Bendzick L., Pribyl L. (2016). Induced pluripotent stem cell-derived natural killer cells for treatment of Ovarian cancer. Stem Cell.

[46] Knorr D.A., Ni Z., Hermanson D. (2013). Clinical-scale derivation of natural killer cells from human pluripotent stem cells for cancer therapy. Stem Cells Transl Med.

[47] Denman C.J., Senyukov V.V., Somanchi S.S. (2012). Membrane-bound IL-21 promotes sustained ex vivo proliferation of human natural killer cells. PLoS One.

[48] Hammill D. (2021).

[49] Crowell H.L., Zanotelli V.R.T., Chevrier S., Robinson M.D. (2022).

[50] Yang C., Siebert J.R., Burns R. (2019). Heterogeneity of human bone marrow and blood natural killer cells defined by single-cell transcriptome. Nat Commun.

[51] Hoffman P. (2022).

[52] R Core Team (2021).

[53] Pliner H., Qiu X., Trapnell C. (2022).

[54] Satija R., Butler A., Hoffman P., Stuart T. (2020).

[55] Oksanen J., Simpson G.L., Blanchet F.G. (2022).

[56] Alquicira-Hernandez J., Powell J.E. (2021). Nebulosa recovers single-cell gene expression signals by kernel density estimation. Bioinformatics.

[57] Bunis D., Andrews J. (2021).

[58] Sievert C., Parmer C., Hocking T. (2021).

[59] Gu Z. (2021).

[60] Gu Z., Eils R., Schlesner M. (2016). Complex heatmaps reveal patterns and correlations in multidimensional genomic data. Bioinformatics.

[61] Wickham H. (2021).

[62] Wickham H., Averick M., Bryan J. (2019). Welcome to the tidyverse. J Open Source Softw.

[63] Wu X., Jin L.P., Yuan M.M., Zhu Y., Wang M.Y., Li D.J. (2005). Human first-trimester trophoblast cells recruit CD56^bright^CD16^−^ NK cells into decidua by way of expressing and secreting of CXCL12/stromal cell-derived factor 1. J Immunol.

[64] Tao Y., Li Y.-H., Piao H.-L. (2015). CD56^bright^CD25^+^ NK cells are preferentially recruited to the maternal/fetal interface in early human pregnancy. Cell Mol Immunol.

[65] Lu H., Jin L.-P., Huang H.-L. (2020). Trophoblast-derived CXCL12 promotes CD56^bright^ CD82^−^CD29^+^ NK cell enrichment in the decidua. Am J Reprod Immunol.

[66] López-Soto A., Gonzalez S., Smyth M.J., Galluzzi L. (2017). Control of metastasis by NK cells. Cancer Cell.

[67] Crouse J., Xu H.C., Lang P.A., Oxenius A. (2015). NK cells regulating T cell responses: mechanisms and outcome. Trends Immunol.

[68] Moffett-King A. (2002). Natural killer cells and pregnancy. Nat Rev Immunol.

[69] Michel T., Poli A., Cuapio A. (2016). Human CD56^bright^ NK cells: an update. J Immunol.

[70] Bernardini G., Gismondi A., Santoni A. (2012). Chemokines and NK cells: regulators of development, trafficking and functions. Immunol Lett.

[71] Abel A.M., Yang C., Thakar M.S., Malarkannan S. (2018). Natural killer cells: development, maturation, and clinical utilization. Front Immunol.

[72] Berahovich R.D., Lai N.L., Wei Z., Lanier L.L., Schall T.J. (2006). Evidence for NK cell subsets based on chemokine receptor expression. J Immunol.

[73] Inngjerdingen M., Damaj B., Maghazachi A.A. (2001). Expression and regulation of chemokine receptors in human natural killer cells. Blood.

[74] Esche C., Stellato C., Beck L.A. (2005). Chemokines: key players in innate and adaptive immunity. J Invest Dermatol.

[75] Fehniger T.A., Cooper M.A., Nuovo G.J. (2003). CD56^bright^ natural killer cells are present in human lymph nodes and are activated by T cell–derived IL-2: a potential new link between adaptive and innate immunity. Blood.

[76] Borg C., Jalil A., Laderach D. (2004). NK cell activation by dendritic cells (DCs) requires the formation of a synapse leading to IL-12 polarization in DCs. Blood.

[77] Devêvre E.F., Renovato-Martins M., Clément K., Sautès-Fridman C., Cremer I., Poitou C. (2015). Profiling of the three circulating monocyte subpopulations in human obesity. J Immunol.

[78] Hyun Y.-M., Hong C.-W. (2017). Deep insight into neutrophil trafficking in various organs. J Leukoc Biol.

[79] Sohlberg E., Pfefferle A., Heggernes Ask E. (2022). Perturbed NK-cell homeostasis associated with disease severity in chronic neutropenia. Blood.

[80] Juelke K., Killig M., Luetke-Eversloh M. (2010). CD62L expression identifies a unique subset of polyfunctional CD56^dim^ NK cells. Blood.

[81] Zhang J., Endres S., Kobold S. (2019). Enhancing tumor T cell infiltration to enable cancer immunotherapy. Immunotherapy.

[82] Kim C.H., Rott L., Kunkel E.J. (2001). Rules of chemokine receptor association with T cell polarization in vivo. J Clin Invest.

[83] Bonecchi R., Bianchi G., Bordignon P.P. (1998). Differential expression of chemokine receptors and chemotactic responsiveness of type 1 T helper cells (Th1s) and Th2s. J Exp Med.

[84] Lintermans L.L., Rutgers A., Stegeman C.A., Heeringa P., Abdulahad W.H. (2017). Chemokine receptor co-expression reveals aberrantly distributed TH effector memory cells in GPA patients. Arthritis Res Ther.

[85] Yamamoto J., Adachi Y., Onoue Y. (2000). Differential expression of the chemokine receptors by the Th1- and Th2-type effect or populations within circulating CD4^+^ T cells. J Leukoc Biol.

[86] Campbell J.D., HayGlass K.T. (2000). T cell chemokine receptor expression in human Th1-and Th2-associated diseases. Arch Immunol Ther Exp.

[87] Bachelerie F., Ben-Baruch A., Burkhardt A.M. (2014). International Union of Basic and Clinical Pharmacology. LXXXIX. Update on the extended family of chemokine receptors and introducing a new nomenclature for atypical chemokine receptors. Pharmacol Rev.

[88] Sandblad K.G., Jones P., Kostalla M.J., Linton L., Glise H., Winqvist O. (2015). Chemokine receptor expression on monocytes from healthy individuals. Clin Immunol.

[89] Zhao X., Gu M., Xu X. (2020). CCL3/CCR1 mediates CD14^+^CD16^−^ circulating monocyte recruitment in knee osteoarthritis progression. Osteoarthritis Cartilage.

[90] Martínez Muñoz L., Lucas P., Navarro G. (2009). Dynamic regulation of CXCR1 and CXCR2 homo- and heterodimers1. J Immunol.

[91] Klanova M., Oestergaard M.Z., Trněný M. (2019). Prognostic impact of natural killer cell count in follicular lymphoma and diffuse large B-cell lymphoma patients treated with immunochemotherapy. Clin Cancer Res.

[92] Kim J.-K., Chung J.-S., Shin H.-J. (2014). Influence of NK cell count on the survival of patients with diffuse large B-cell lymphoma treated with R-CHOP. Blood Res.

[93] Saetersmoen M.L., Hammer Q., Valamehr B., Kaufman D.S., Malmberg K.-J. (2019). Off-the-shelf cell therapy with induced pluripotent stem cell-derived natural killer cells. *Seminars in immunopathology. Springer;*.

