# Supplementary material for: Mapping the chemotactic landscape in NK cells reveals subset-specific synergistic migratory responses to dual chemokine receptor ligation
Source: eBioMedicine. 2023 Sep 21;96:104811. doi: 10.1016/j.ebiom.2023.104811 (PMC10520535; doi:10.1016/j.ebiom.2023.104811)
Supplement: Supplementary Data [file mmc1.docx]

Mapping the Chemotactic Landscape in NK Cells Reveals Subset-specific Synergistic Migratory Responses to Dual Chemokine Receptor Ligation

Mieszko Lachota^1,2^, Katarzyna Zielniok^1^, Daniel Palacios^3^, Minoru Kanaya^3^, Leena Peena^4^, Hanna Julie Hoel^3^, Merete Thune Wiiger^3^, Lise Kveberg^3^, Wojciech Hautz^2^, Radosław Zagożdżon^1^, Karl-Johan Malmberg^3,5*^

^1^ Department of Clinical Immunology, Medical University of Warsaw, Warsaw, Poland

^2^ Department of Ophthalmology, Children’s Memorial Health Institute, Warsaw, Poland

^3^ Institute for Cancer Research, Department of Cancer Immunology, University of Oslo, Oslo University Hospital, Norway.

^4^ Finnish Red Cross Blood Service, Research and Development, Helsinki, Finland.

^5^ Center for Infectious Medicine, Department of Medicine Huddinge, Karolinska Institutet, Stockholm, Sweden

Supplementary Data

| **Fluorochrome** | **Antigen** | **Clone** | **Dilution** | **Vendor** |
| --- | --- | --- | --- | --- |
| **V500** | CD14 | M5E2 | 1:50 | BD Bioscience |
| **V500** | CD19 | HIB19 | 1:50 | BD Bioscience |
| **VioGreen** | CD3 | REA613 | 1:50 | Miltenyi |
| **Biotin** | CD56 | REA196 | 1:50 | Miltenyi |
| **APC-Vio770** | CD57 | REA769 | 1:50 | Miltenyi |
| **BV786** | NKG2A | 131411 | 1:50 | BD Bioscience |
| **APC** | NKG2A | Z199 | 1:25 | Beckman Coulter |
| **AF700** | NKG2C | 134591 | 1:50 | R&D |
| **PE** | NKG2C | REA205 | 1:50 | Miltenyi |
| **PerCp** | KIR2DL3 | REA147 | 1:50 | Miltenyi |
| **FITC** | KIR2DL1 | REA284 | 1:50 | Miltenyi |
| **BV421** | KIR3DL1 | REA1005 | 1:50 | Miltenyi |
| **PerCp** | KIR2DL2/L3/S2 | GL183 | 1:25 | Beckman Coulter |
| **PE-Vio770** | KIR2DL1/S1 | REA1010 | 1:10 | Miltenyi |
| **AF700** | Granzyme B | GB11 | 1:100 | BD Bioscience |
| **PE** | CD62L | DREG-56 | 1:50 | Biolegend |
| **PE** | CD162 | KPL-1 | 1:50 | Biolegend |
| **PE** | CD50 | CRB-IC3/1 | 1:50 | Biolegend |
| **PE** | Integrin B71 | FIB504 | 1:50 | Biolegend |
| **PE** | CD11b | M1/70 | 1:50 | Biolegend |
| **PE** | CD18 | TS1/18 | 1:50 | Biolegend |
| **APC** | CCRL2 | 152254 | 1:50 | Novus |
| **PE & APC** | CCR1 | REA158 | 1:50 | Miltenyi |
| **PE & APC** | CCR2 | REA264 | 1:50 | Miltenyi |
| **PE & VioBlue** | CCR3 | REA574 | 1:50 | Miltenyi |
| **PE & APC** | CCR4 | REA279 | 1:50 | Miltenyi |
| **PE & APC** | CCR5 | REA245 | 1:50 | Miltenyi |
| **PE & APC** | CCR6 | REA190 | 1:50 | Miltenyi |
| **PE & APC** | CCR7 | REA108 | 1:50 | Miltenyi |
| **PE** | CCR8 | L263G8 | 1:50 | Biolegend |
| **PE & APC** | CCR9 | REA469 | 1:50 | Miltenyi |
| **PE & APC** | CCR10 | REA326 | 1:50 | Miltenyi |
| **PE & VioBlue** | CXCR1 | REA958 | 1:50 | Miltenyi |
| **PE & APC** | CXCR2 | REA208 | 1:50 | Miltenyi |
| **PE & APC** | CXCR3 | REA232 | 1:50 | Miltenyi |
| **PE & APC** | CXCR4 | REA649 | 1:50 | Miltenyi |
| **PE & APC** | CXCR5 | REA103 | 1:50 | Miltenyi |
| **PE & APC** | CXCR6 | REA458 | 1:50 | Miltenyi |
| **PE & APC** | CX3CR1 | 2A9-1 | 1:25 | Miltenyi |
| **PE & APC** | CMKLR1 | REA455 | 1:50 | Miltenyi |
| **PE** | XCR1 | 1097A | 1:50 | R&D |

Supplementary Table S1. The list of antibodies used in flow cytometry experiments.

| **Mass** | **Antigen** | **Clone** | **Dilution** | **Vendor** |  |
| --- | --- | --- | --- | --- | --- |
| **89Y** | CD45 | HI30 | 1:400 | Fluidigm |  |
| **141Pr** | CX3CR1 | REA385 | 1:100 | Miltenyi |  |
| **142Nd** | CD57 | HCD57 | 1:1600 | Fluidigm |  |
| **143Nd** | CD2 | TS1/8 | 1:800 | Biolegend | |
| **144Nd** | CD38 | REA572 | 1:800 | Miltenyi |  |
| **145Nd** | CXCR3 | REA232 | 1:50 | Miltenyi |  |
| **146Nd** | CCR2 | REA538 | 1:50 | Miltenyi |  |
| **147Sm** | CD96 | NK92.39 | 1:100 | Biolegend |  |
| **148Nd** | CD3 | OKT3 | 1:800 | Biolegend |  |
| **148Nd** | CD14 | RMO52 | 1:400 | Fluidigm |  |
| **148Nd** | CD19 | HIB19 | 1:400 | Biolegend |  |
| **149Sm** | FasL | NOK-1 | 1:100 | Biolegend |  |
| **150Nd** | LAG3 | 11C3C65 | 1:200 | Fluidigm |  |
| **151Eu** | CXCR4 | REA649 | 1:200 | Miltenyi |  |
| **152Sm** | Siglec-7 | 194211 | 1:100 | Fluidigm |  |
| **153Eu** | TIM-3 | F38-2E2 | 1:100 | Fluidigm |  |
| **154Sm** | NKG2C | REA205 | 1:1600 | Miltenyi |  |
| **155Gd** | CXCR1 | REA958 | 1:200 | Miltenyi |  |
| **156Gd** | KIR2DL1/S1 | 11PB6 | 1:100 | Miltenyi |  |
| **158Gd** | KIR2DL1 | REA284 | 1:50 | Miltenyi |  |
| **159Tb** | GITR | 108-17 | 1:200 | Biolegend |  |
| **160Gd** | GPR56 | CG4 | 1:600 | Biolegend |  |
| **161Dy** | PLZF | 17.10,.17 | 1:100 | Biolegend |  |
| **162Dy** | CD69 | FN50 | 1:200 | Fluidigm |  |
| **163Dy** | KIR2DL2/S2/L3 | GL183 | 1:400 | Beckman Coulter |  |
| **164Dy** | TIGIT | MBSA43 | 1:200 | Thermo Fisher |  |
| **165Ho** | CCR5 | REA245 | 1:800 | Miltenyi |  |
| **166Er** | NKG2D | ON72 | 1:100 | Fluidigm |  |
| **167Er** | CCR7 | REA108 | 1:100 | Miltenyi |  |
| **168Er** | NKp30 | P30-15 | 1:100 | Miltenyi |  |
| **169Tm** | NKG2A | Z199 | 1:200 | Fluidigm |  |
| **170Er** | CXCR2 | REA208 | 1:50 | Miltenyi |  |
| **171Yb** | DNAM-1 | DX11 | 1:200 | Fluidigm |  |
| **172Yb** | KIR3DL1 | DX9 | 1:800 | R&D |  |
| **173Yb** | Granzyme B | GB11 | 1:400 | Fluidigm |  |
| **174Yb** | PD-1 | EH12.2H7 | 1:100 | Fluidigm |  |
| **175Lu** | Perforin | B-D48 | 1:400 | Fluidigm |  |
| **176Yb** | CD56 | NCAM16.2 | 1:400 | Fluidigm |  |
| **209Bi** | CD16 | 3G8 | 1:200 | Fluidigm |  |

Supplementary Table S2. The list of antibodies used in mass cytometry experiment.


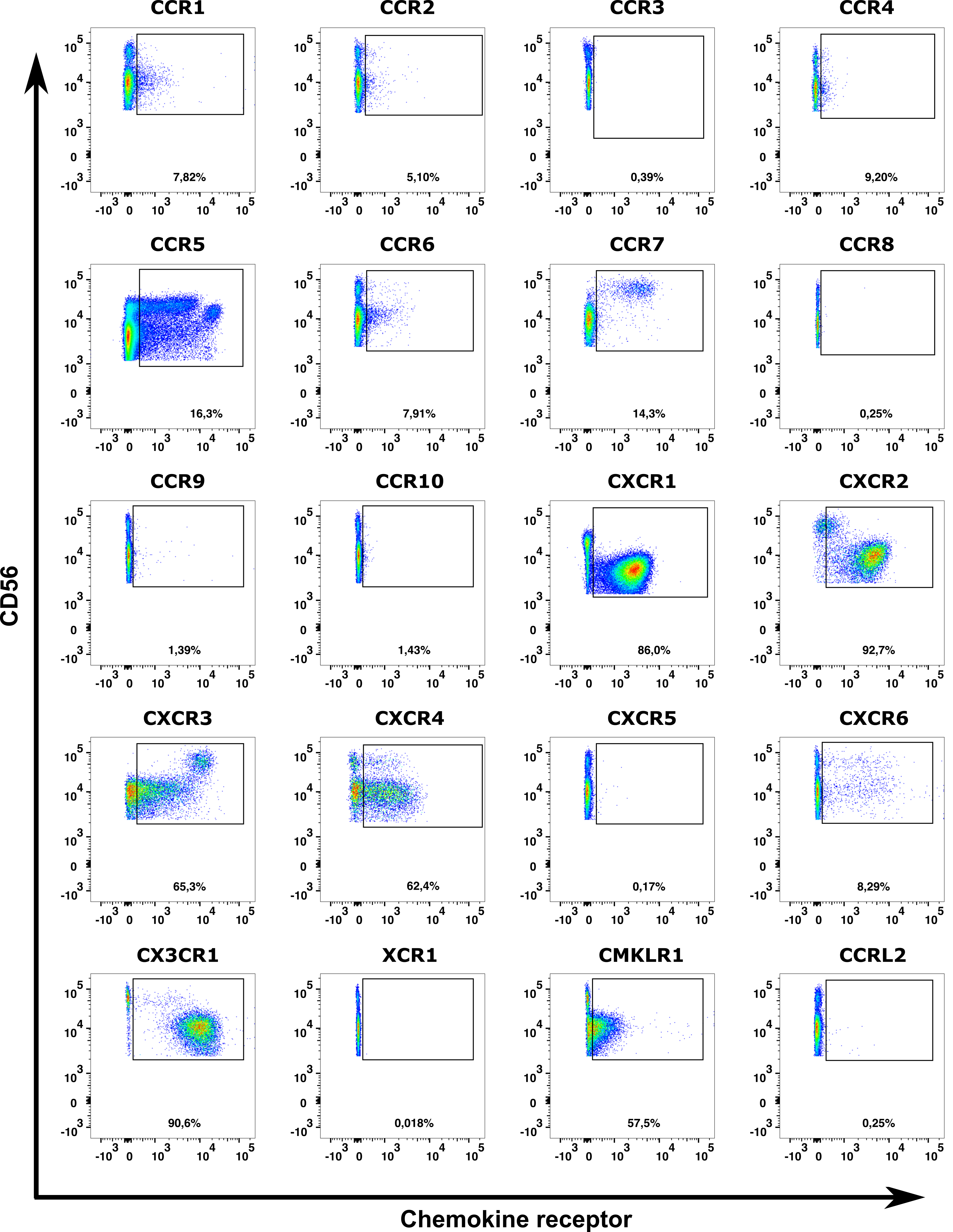


Supplementary Figure S1. Representative staining of chemokine receptors on peripheral blood NK cells.

NK cells were identified as live, single, CD56+ CD3-, CD14-, CD19- cells in peripheral blood mononuclear cells isolated from healthy donors. Each plot represents expression of chemokine receptor (X-axis) and CD56 (Y-axis).

Supplementary Figure S2. Chemokine receptor expression in NK cells.

NK cells in PBMC, after isolation, with and without different stimuli were profiled for chemokine receptor expression. Chemokine receptor expression is represented as percentage of cells expressing given chemokine receptor. Each experiment (n=3) was performed on independent donors. Each dot represents each experiment/donor.


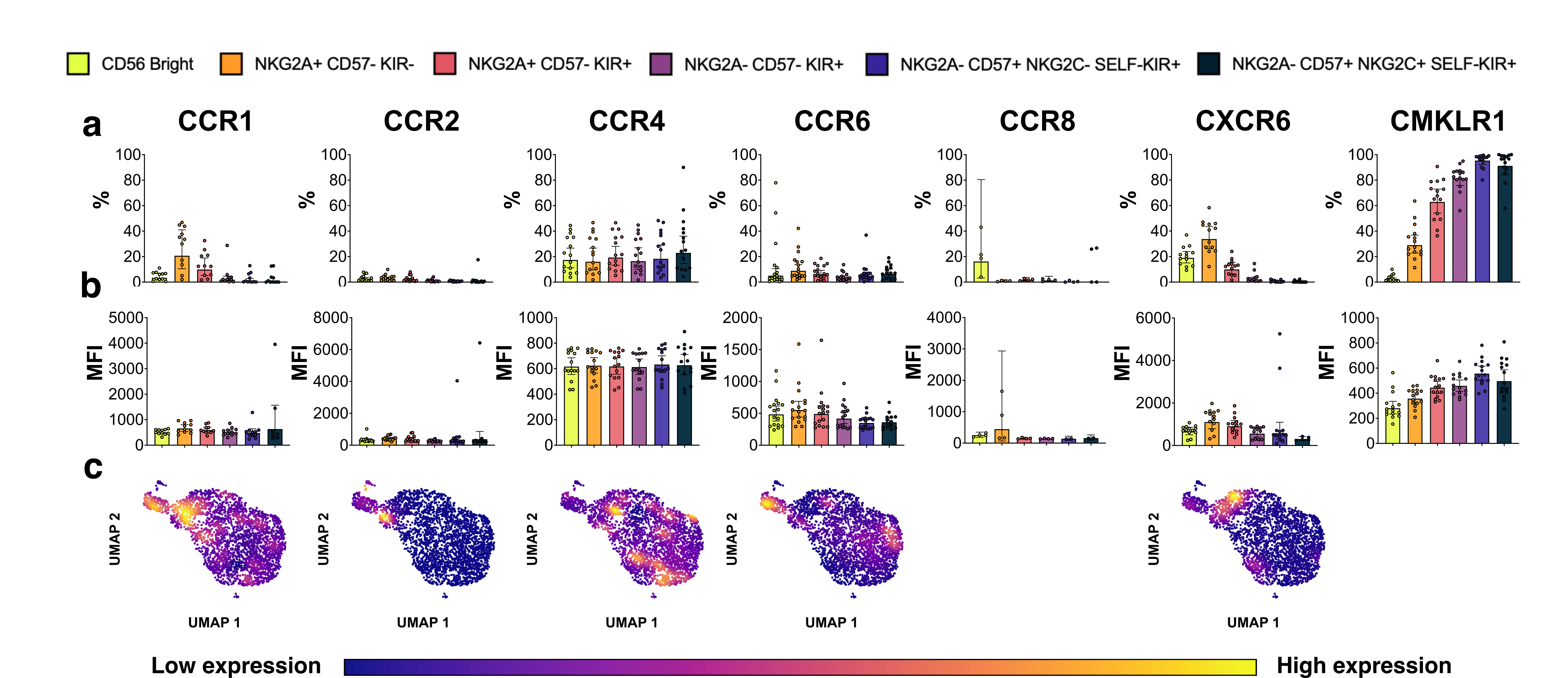


Supplementary Figure S3. The effects of NK cell differentiation on expression of other chemokine receptors.

NK cell subsets were identified by CD56, NKG2A, CD57, KIR and NKG2C expression and ordered accordingly to their differentiation status. Each heading applies to the whole column. The legend applies to sections A and B where each dot represents an individual donor. Bars in (A) and (B) represent geometric mean with 95% confidence interval. Two technical replicates were assessed in each donor. The chemokine receptor expression is presented as (A) percent of cells expressing a marker (B) median fluorescent intensity (C) chemokine receptor mRNA expression derived from single-cell RNA-seq data. The single-cell RNA-seq clustering and pseudotime analysis is presented in Supp. Fig. 5 & 9. The cells are positioned from the least mature (left, top) to most mature (right, bottom).


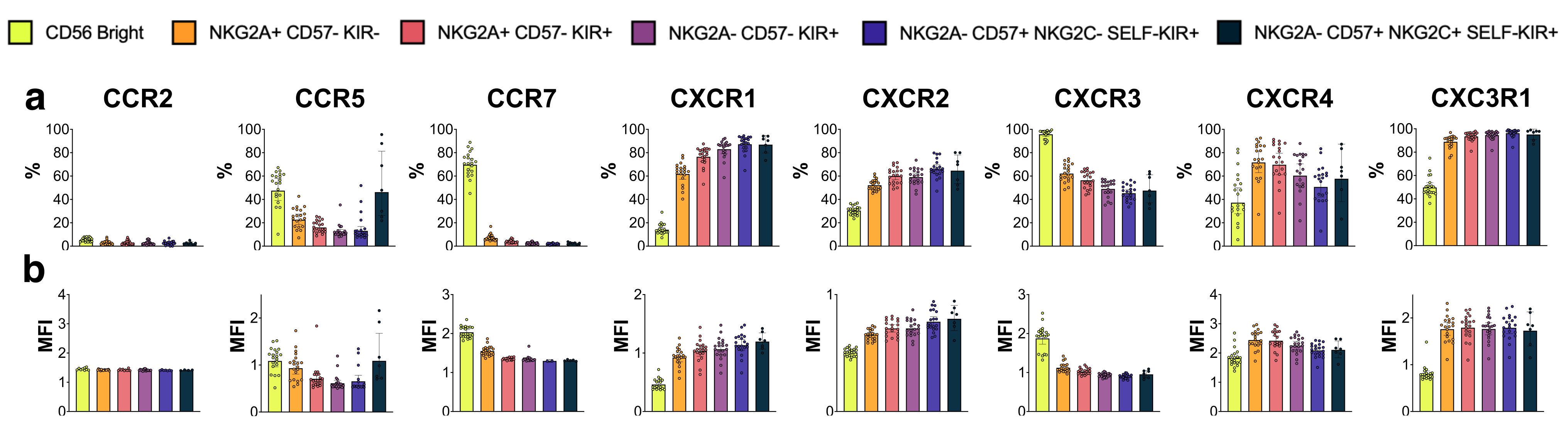


Supplementary Figure S4. The effects of NK cell differentiation on expression of chemokine receptors verified by mass cytometry.

The experiment was performed on cryopreserved PBMC samples from 20 healthy donors. NK cell subsets were identified in the same gating scheme as in Figure 2. Briefly, NK cell subsets were identified by CD56, NKG2A, CD57, KIR and NKG2C expression and ordered accordingly to their differentiation status. Each heading applies to the whole column. The chemokine receptor expression is presented as (A) percent of cells expressing a marker (B) median staining intensity. Each dot represents an individual donor. Bars represent geometric mean with 95% confidence interval.


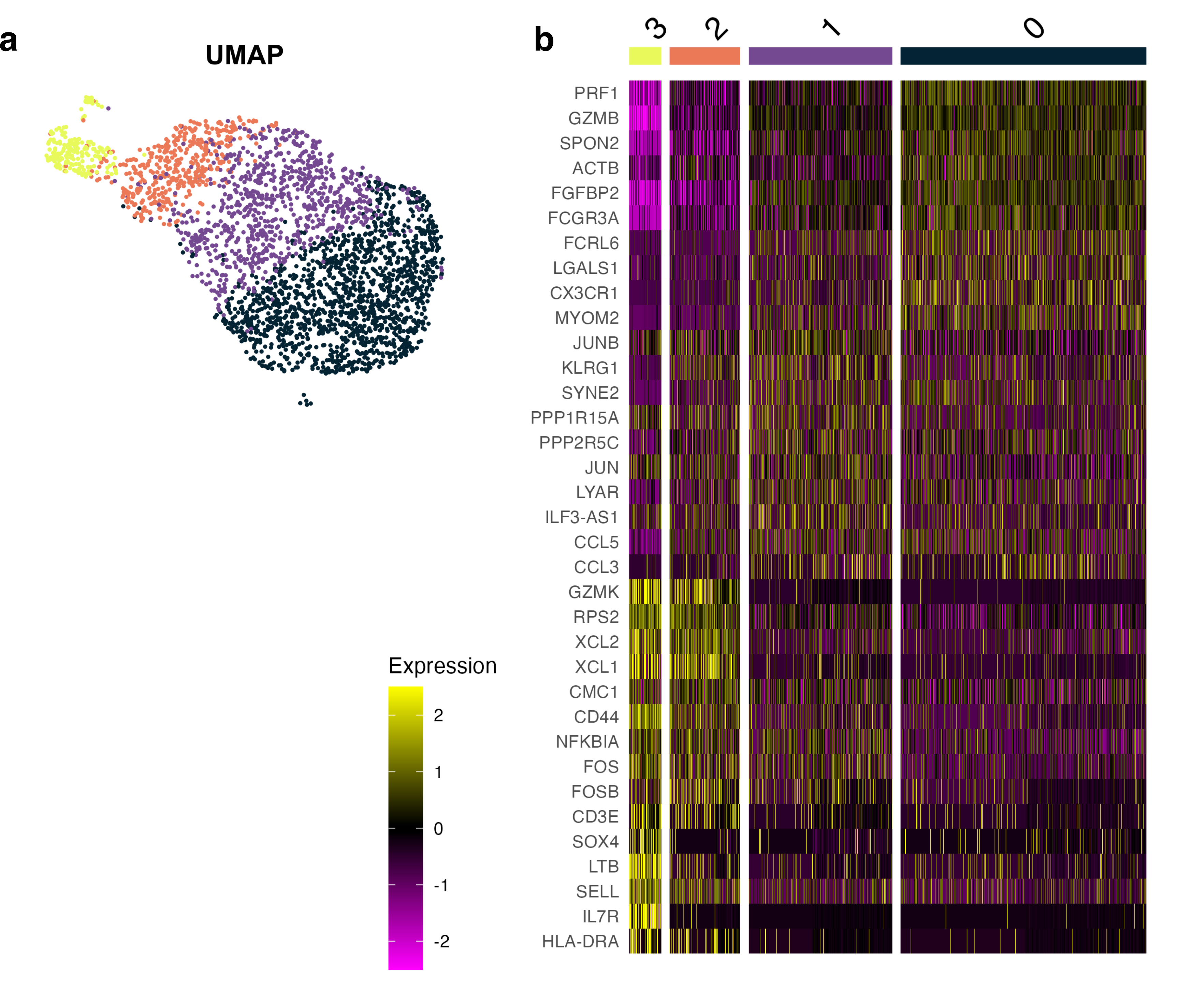


Supplementary Figure S5. Unbiased clustering of human peripheral blood NK cells.

**A.** Four distinct human PB NK clusters were numbered and displayed with an UMAP plot **B.** Top 10 up-regulated DEGs (ranked by log fold change) of each cluster are presented as a heatmap.

**
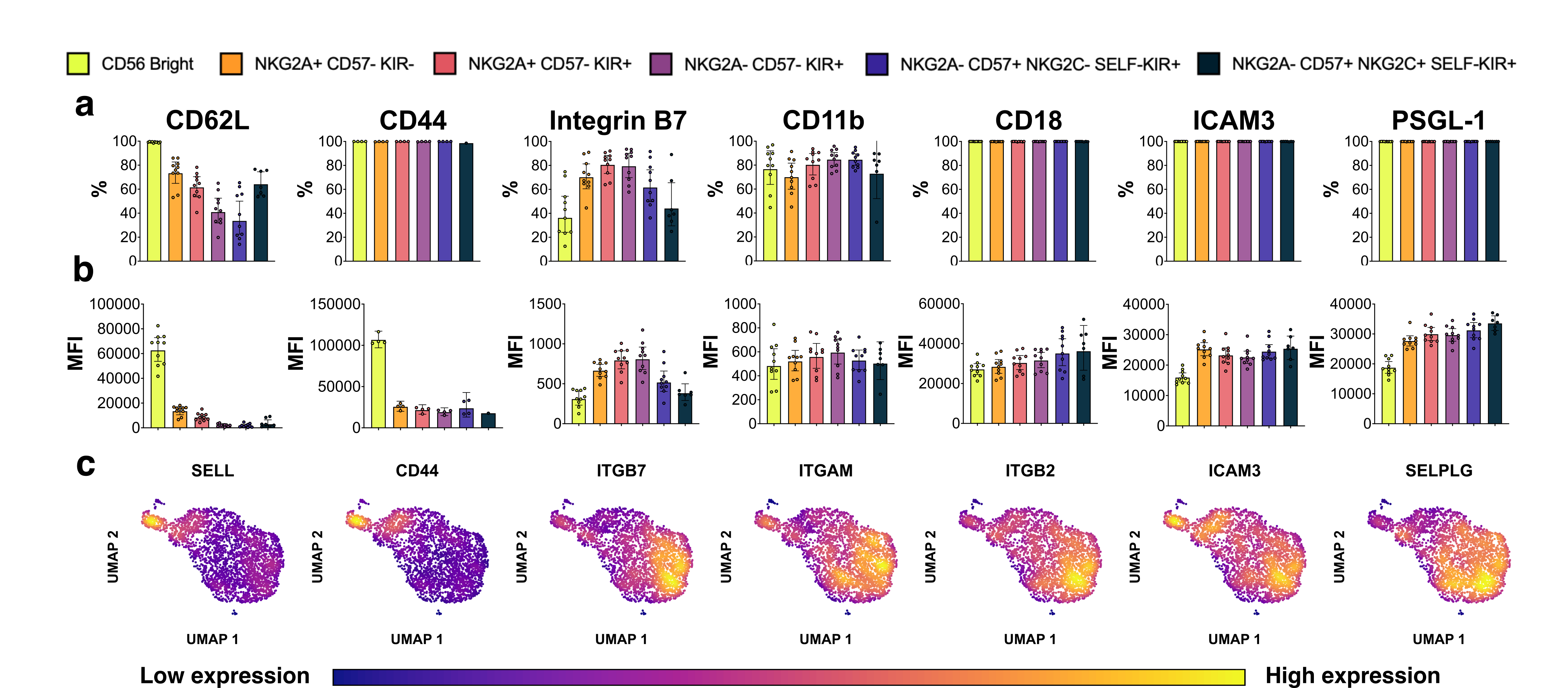
**

Supplementary Figure S6. The effects of differentiation on NK cell adhesion molecule expression.

NK cell subsets were identified by CD56, NKG2A, CD57, KIR and NKG2C expression and ordered accordingly to their differentiation status. Each heading applies to the whole column. The legend applies to sections A and B where each dot represents an individual donor. Bars in (A) and (B) represent geometric mean with 95% confidence interval. Two technical replicates were assessed in each donor. The adhesion molecule expression is presented as (A) percent of cells expressing a marker (B) median fluorescent intensity (C) adhesion molecule mRNA expression derived from single-cell RNA-seq data. The single-cell RNA-seq clustering and pseudotime analysis is presented in Supp. Fig. 5 & 9. The cells are positioned from the least mature (left, top) to most mature (right, bottom).


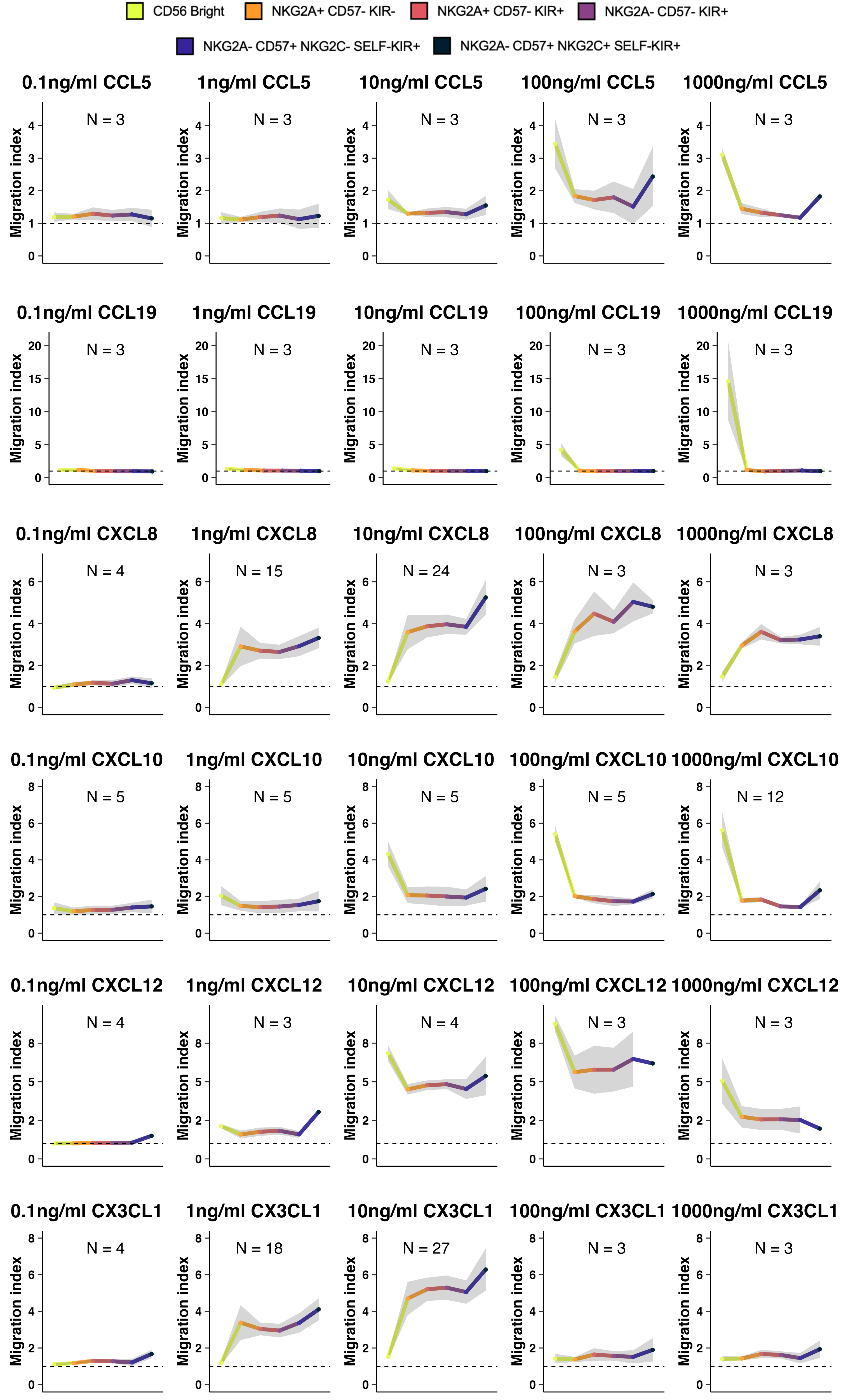

Supplementary Figure S7. Chemokine titration in NK cell subsets

A transwell-based titration of different chemokines in a subset resolution. Migration index was calculated as a ratio of cells that have migrated to the bottom well during chemokine-induced migration compared to the cells that have spontaneously migrated to the bottom well, without any chemoattractant present. NK cell subsets were identified by CD56, NKG2A, CD57, NKG2C, and KIR expression, and ordered accordingly to their differentiation status. “N” specifies number of donors in each condition. Data represents means ± SEM (gray area). Some chemokines (e.g., CX3CL1, CXCL8) in some concentrations (e.g., 1ng/ml, 10ng/ml) were evaluated in more donors as they were used for the further experiments evaluating potential synergies (Figure 3).

Supplementary Figure S8. Synergistic or additive effects of chemokine receptor simultaneous ligation?

Analysis of synergistic effects between two chemokines at the subset resolution. The migration index was calculated as a ratio of cells that have migrated to the bottom well during chemokine-induced migration compared to the cells that have spontaneously migrated to the bottom well, without any chemoattractant present. NK cell subsets were identified by CD56, NKG2A, CD57, NKG2C, and KIR expression, and ordered accordingly to their differentiation status. The experiment was performed on ten donors. Data represent**s** geometric mean. The differences between the curves representing 1ng/ml CX3CL1 & 1ng/ml CXCL8 and the mathematic addition of migration index of 1ng/ml CX3CL1 and 1ng/ml CXCL8 was assessed for each subset using the Wilcoxon test, which revealed statistically significant difference in adaptive CD56^dim^ NKG2A^-^ CD57^+^ NKG2C^+^ self-KIR^+^ NK cell subset.


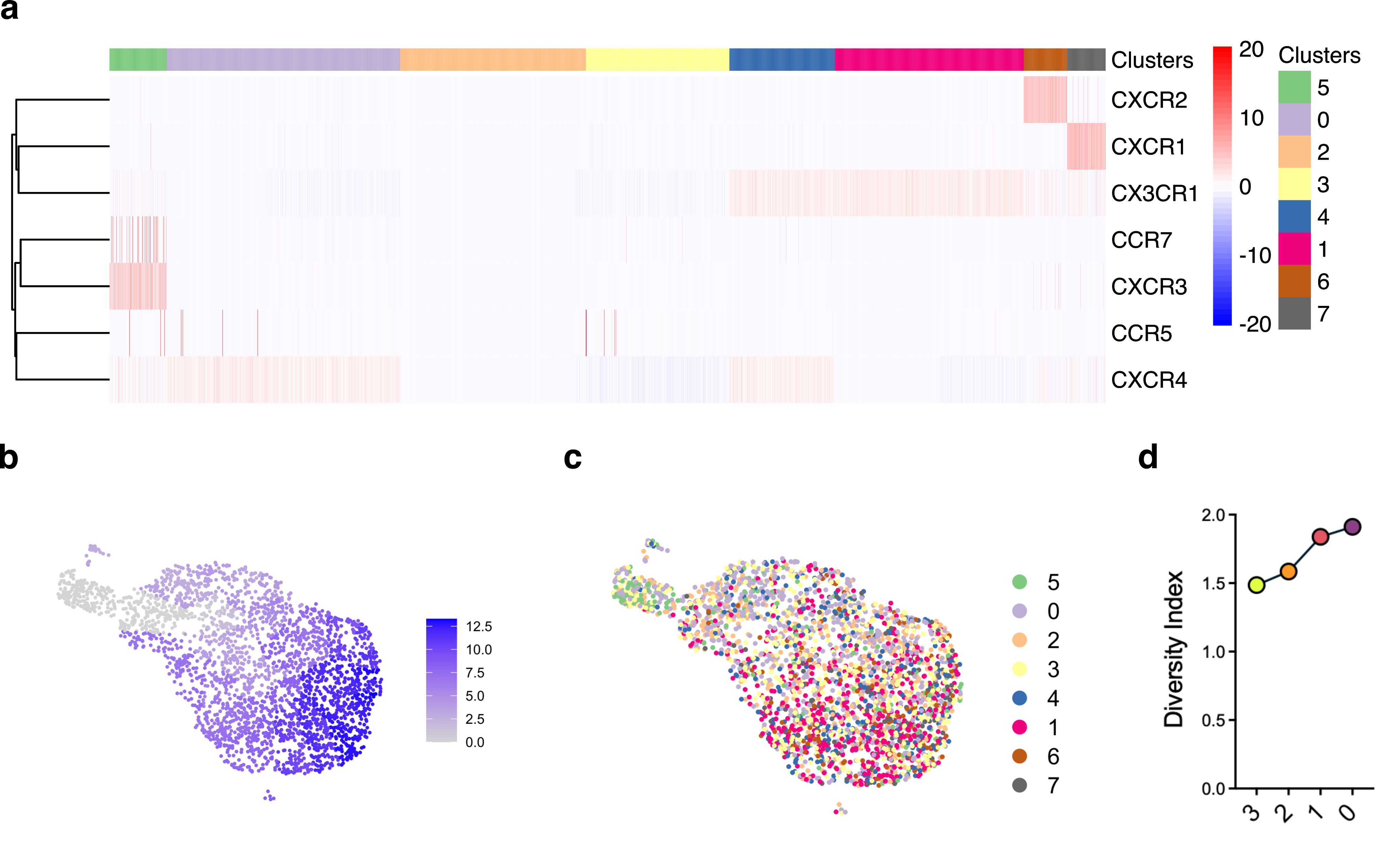


Supplementary Figure S9. Chemokine receptor-based clustering of peripheral blood NK cells.

The results of clustering peripheral blood NK cells based on expression of 7 chemokine receptors: CCR5, CCR7, CXCR1, CXCR2, CXCR3, CXCR4 and CX3CR1. **A.** Distribution of chemokine receptors in each cluster. **B.** Pseudotime visualizing developmental trajectories assigned to each cell in UMAP plot. **C.** Chemokine-receptor clustering results visualized as identity-colored UMAP plot. **D.** Chemokine receptor diversity calculated with Shannon index in NK cell differentiation-based clusters.
